# Efficacy and Safety of “Three Chinese Patent Medicines and Three TCM Prescriptions” for COVID-19: A Systematic Review and Network Meta-Analysis

**DOI:** 10.1155/2022/4654793

**Published:** 2022-01-12

**Authors:** Shuo Zhang, Zhen Yang, Zhen-Lin Chen, Zhuo-Ning Li, Shi-Jun Yue, Jia-Jia Li, Yu-Ping Tang

**Affiliations:** ^1^Key Laboratory of Shaanxi Administration of Traditional Chinese Medicine for TCM Compatibility, State Key Laboratory of Research & Development of Characteristic Qin Medicine Resources (Cultivation), Shaanxi Key Laboratory of Chinese Medicine Fundamentals and New Drugs Research, Shaanxi Collaborative Innovation Center of Chinese Medicinal Resources Industrialization, Shaanxi University of Chinese Medicine, Xi'an 712046, Shaanxi Province, China; ^2^School of Clinical Medicine (Guang'anmen Hospital), Beijing University of Chinese Medicine, Beijing 100029, China; ^3^School of Public Health, Shaanxi University of Chinese Medicine, Xi'an 712046, Shaanxi Province, China; ^4^International Programs Office, Shaanxi University of Chinese Medicine, Xi'an 712046, Shaanxi Province, China

## Abstract

**Objective:**

To systematically evaluate the efficacy, safety, and precision of TMTP for COVID-19.

**Methods:**

Randomized controlled trials and retrospective studies were searched in 11 electronic databases. This network meta-analysis included trials using TMTP to treat patients with COVID-19. The traditional pairwise meta-analysis was done by using Stata 15, and Bayesian network meta-analysis was done with WinBUGS.

**Results:**

18 trials were included with 2036 participants and 7 drugs. The results showed that LHQW had the most significant effects on improving expectoration, shortness of breath, sore throat, nausea, emesis, inappetence, muscle soreness, and headache, and it could produce the least adverse reactions. XBJ was the best drug for fever, fatigue, and diarrhea, which showed great advantages in lowering WBC levels. XFBD was the most effective drug for cough and chest distress, which had the least exacerbation rate. JHQG was the most effective for rhinobyon and rhinorrhea, while QFPD was the best drug in decreasing CRP levels.

**Conclusion:**

This study was the first most large-scale and comprehensive research of TMTP for COVID-19. The results showed that LHQW had good efficacy without obvious adverse reactions. Therefore, we believe that it should be firstly recommended for COVID-19 treatment. In addition, XBJ is recommended for patients with a severe fever, fatigue, and diarrhea, and JHQG is recommended for patients with obvious rhinobyon and rhinorrhea; then, XFBD is recommended for patients with cough and chest tightness as the main manifestation. Our findings will help experts develop new COVID-19 treatment guidelines to better guide clinical medication for protecting the health of COVID-19 patients.

## 1. Introduction

A severe respiratory disease caused by a novel coronavirus infection broke out in late 2019. Since its outbreak, the Corona Virus Disease 2019 (COVID-19) has spread rapidly and enveloped most of the world which is highly contagious and deadly [1]. The increase in the number of pneumonia patients has aroused high vigilance from the World Health Organization (WHO) and China [2] and has been listed as a public health emergency of international concern. As of 8 December 2021, COVID-19 has caused 267534728 confirmed cases and 5290066 deaths worldwide. The COVID-19 pandemic has not only posed a huge challenge to medical workers but also caused social, economic, and political damage. Although a large number of clinical trials have been conducted to study the drugs that were used to treat COVID-19, no therapeutic approach has been effective in its treatment up to now [3]. Moreover, during the sudden outbreak of COVID-19, there is a lack of effective chemical drugs (CDs) for prevention and control in clinical practice, and the interim screening and development of new drugs may take a long time, making it difficult to apply them in a timely way for clinical treatment. Therefore, the unique advantages of traditional Chinese medicine (TCM) multitarget interventions had become an indispensable systemic approach for patients, and significant progress has been made in China's battle against COVID-19 in China [4].

TCM has been proven to be effective in treating patients with influenza and has very successful experience in the prevention and treatment of infectious diseases. Three Chinese patent medicines and three TCM prescriptions (TMTP) were the key recommended drugs in the *Novel Coronavirus Infection for the Diagnosis and Treatment of Pneumonia* published by the National Health Commission of the People's Republic of China. And the China Administration of Traditional Chinese Medicine pointed out that TMTP had a great clinical advantage in the prevention and control of COVID-19. In addition, three Chinese patent medicines included *Lian-Hua-Qing-Wen* (LHQW), *Jin-Hua-Qing-Gan* (JHQG), and *Xue-Bi-Jing* (XBJ), and three TCM prescriptions included *Qing-Fei-Pai-Du* (QFPD), *Xuan-Fei-Bai-Du* (XFBD), and *Hua-Shi-Bai-Du* (HSBD), which had played an important role in the fight against the epidemic by treating the disease based on syndrome differentiation according to the patient's individuality and seasonal and local conditions. Although TMTP was widely used in clinical practice, there were inconsistent research conclusions due to the different sample sizes, outcome indicators, and unified evaluation criteria in clinical reports, and accurate conclusions cannot be drawn, which made it difficult to grasp its therapeutic effect and clinical advantages. Therefore, we conducted a comprehensive systematic review and meta-analysis to evaluate TMTP efficacy and safety of COVID-19 patients from a variety of clinical symptoms, in order to serve for COVID-19 prevention and control better.

It is a challenging task to comprehensively analyze the present clinical evidence for the efficacy and safety of TMTP for COVID-19 by using traditional meta-analysis methods because there is a lack of head-to-head trials that directly compare certain treatments among existing trials. Bayesian network meta-analysis, also known as a mixed treatment comparison method, is a valuable tool in comparative effectiveness research, which enables the comparison of multiple interventions to incorporate clinical evidence from both direct and indirect treatment comparisons in a network of treatments and associated trials [5], and allows indirect comparison without head-to-head trials to simultaneously compare several treatments by using a common comparator and combined direct and indirect comparisons while retaining randomness in individual trials [6–8].

This study aimed to systematically evaluate the efficacy and safety of TMTP for COVID-19 using Bayesian network meta-analysis, and the competing drugs in each outcome were ranked to obtain the most effective one. The study focused on the diversity of symptoms in patients with COVID-19, which was the most large-scale and comprehensive research on this issue so far. We hope that our study will provide up-to-date information on the treatment of COVID-19.

## 2. Data and Methods

This systematic review and Bayesian network meta-analysis was conducted and reported following the Preferred Reporting Items for Systematic Reviews and Meta-analyses (PRISMA) with Cochrane methodology [9]. This study has been registered and the PROSPERO number is CRD42021240869.

### 2.1. Literature Search

From the establishment of each electronic database to November 6, 2021, randomized controlled trials or retrospective studies using TMTP to treat COVID-19 were searched in the following 11 electronic databases: Medline (from 1946 to 2020), Embase (from 1974 to 2020), Cochrane Library (from 1966 to 2020), PubMed (from 1959 to 2020), Web of Science (from 1986 to 2020), SpringerLink (from 1996 to 2020), ClinicalTrials.gov, the Chinese National Knowledge Infrastructure (CNKI, from 1980 to 2020), Chinese Biomedical Literature Database (CBM, from 1978 to 2020), Wanfang Database (from 1998 to 2020), and Weipu Database (from 1989 to 2020). Forward and backward citation searching was conducted for all eligible trials. Following terms were used for searching: (“COVID-19” OR “corona virus disease 2019” OR “coronavirus disease 2019” OR “severe acute respiratory syndrome coronavirus” OR “SARS-CoV-2” OR “novel corona virus” OR “novel coronavirus” OR “2019-nCoV” OR “nCoV-2019”) AND (“lianhuaqingwen” OR “lianhua qingwen” OR “lian hua qing wen”) OR (“jinhuaqinggan” OR “jinhua qinggan” OR “jin hua qing gan”) OR (“xuebijing” OR “xue bi jing”) OR (“qingfeipaidu” OR “qingfei paidu” OR “qing fei pai du”) OR (“xuanfeibaidu” OR “xuanfei baidu” OR “xuan fei bai du”) OR (“huashibaidu” OR “huashi baidu” OR “hua shi bai du”) AND (“clinical trial” OR “randomized controlled trial” OR “randomized controlled trial” OR “lin chuang yan jiu” OR “lin chuang shi yan”). And we did not specify the language and status of publications in our literature search. Additionally, we manually searched bibliographies of included trials and related reviews for additional references.

### 2.2. Criteria for Literature Inclusion

#### 2.2.1. Type of Research

This network meta-analysis included clinical trials using TMTP to treat COVID-19. Trials were excluded if (a) no control group was used; (b) TMTP was not used in the experimental group; (c) there is a combination with other drugs; (d) trials on effective analysis data cannot be obtained; (e) they are reviews, conference paper, case reports, experience sharing, animal trials, etc.; (f) they are repeatedly published articles and plagiarized trials.

#### 2.2.2. Study Subjects

COVID-19 patients who were not restricted by age, gender, or nationality were eligible for inclusion in this study. Please refer to the “New Coronavirus Pneumonia Diagnosis and Treatment Program” (trial seventh edition) for details on COVID-19 criteria [10].

#### 2.2.3. Preventative Measures

The intervention measures in the experimental group should be TMTP single drug combined with CD, and the control group should be CD.

#### 2.2.4. Results

The primary outcomes were defined as clinical effect and CT recovery rate. Clinical effect was evaluated based on the improvement of clinical symptoms of patients before and after treatment, which could better reflect the therapeutic effect of drugs. Symptoms were classified as significant, effective, or ineffective according to the degree of relief. Moreover, CT played an important role in early screening and disease surveillance for COVID-19, and its changes could significantly reflect the therapeutic effect. The secondary outcomes included nucleic acid negative rate, disappearance rate of primary symptoms (fever, cough, fatigue), disappearance rate of respiratory (expectoration, shortness of breath, chest distress, rhinobyon, rhinorrhea, sore throat), gastrointestinal (nausea, diarrhea, emesis, inappetence), and other symptoms (muscular soreness, headache), inflammatory biomarkers such as C-reactive protein (CRP) and white blood cell (WBC), as well as exacerbation rate and adverse reactions.

### 2.3. Study Selection and Data Extraction

Before starting the screening processes, two researchers who participated in training and calibration exercises independently screened the titles and abstracts of potentially eligible trials that were in duplicate; then, they independently retrieved and reviewed the full text of the possible trials in duplicate based on the inclusion and exclusion criteria and compared their results. If there was disagreement, they agreed through discussion or submitted it to a third party for evaluation. And before the screening process, the third party used a standardized screening form and performed calibration exercises. The screening process was conducted in Endnote X9.

Before the data extraction process began, we conducted various forms of calibration exercises and pilots. Then, according to the inclusion and exclusion criteria mentioned above, the two researchers used standardized tables to independently extract data in duplicate from all eligible trials. In case of disagreement, they agreed through discussion or submitted it to a third party. All the eligible trials were published in English.

For all eligible trials, the researchers extracted data on the following characteristics:

1. The basic information of the study (author's name, title of the study, year of publication, country/region, and publication status)
2. Study characteristics (sample size, source of cases, age, diagnostic criteria, and inclusion and exclusion criteria)
3. Intervention and control measures (dosage form, dose, and duration)
4. Research methodology (random scheme generation, allocation hiding, blind method, incomplete result data, selective reporting, other biases, and loss of follow-up)
5. Outcome measures

### 2.4. Assessment of Literature Quality

The methodological quality of each included study was independently assessed by two reviewers based on 2 tools. The Cochrane collaboration tool has been used to assess the quality of randomized controlled trials, and it comprised the following 7 aspects: random sequence generation, allocation concealment, blind method, incomplete result data, selective reporting, and other biases. The quality assessment results of each item can be divided into three grades: “low risk,” “high risk,” and “unclear.” The risk coefficient is lower because of the more rigorous design and higher methodological quality of each RCT. Newcastle Ottawa Scale (NOS) has been used to assess the quality of retrospective studies. This method includes 3 aspects of evaluation: the selection method, comparability, and contact exposure assessment method of case and control groups. The higher the score is, the greater the quality of the learning is. When necessary, the consensus on this issue was studied with the help of a third party.

### 2.5. Statistical Analysis

The traditional pairwise meta-analysis was done by using Stata 15 (StataCorp, College Station, TX, USA), and Bayesian network meta-analysis was done with WinBUGS version 14.3 (MRC Biostatistics Unit, Cambridge, UK). Both the continuous and dichotomous outcomes were derived from the included trials without any conversion. The dichotomous outcomes were described by relative risk (RR) and 95% confidence interval (CI); in addition, mean difference (MD) and 95% CI were used to describe the effect value of the intergroup comparison. Heterogeneity was determined according to the results of the *I*^2^ test. *I*^2^ < 50% indicated the low heterogeneity of interstudy, and the fixed effect model was adopted. Furthermore, the random effect model was adopted when *I*^2^ > 50% [11], which was also used to generate direct and mixed treatment comparison estimates. Direct estimates of any two interventions were obtained by pooling data from clinical trials that compared the same interventions face to face. And the mixed treatment comparison estimates of interventions were obtained by combining direct clinical trial data for comparative interventions with the indirect estimates between the interventions through a common comparator. Normal prior distributions, noninformative uniform, and 3 different sets of starting values were used to fit the model. At the same time, 4 chains, 2.5 initial values scaling, 20000 tuning iterations, 50000 simulation iterations, and 10 thinning intervals were used to obtain the posterior distributions of model parameters. Subgroup analysis was used to evaluate the therapeutic effects of different drugs. Inverted funnel plots and Egger's regression test were used to determine publication bias when the number of included studies exceeded 10 in the network meta-analysis [12].

## 3. Results

### 3.1. Results of Our Literature Search

**Figure 1:**
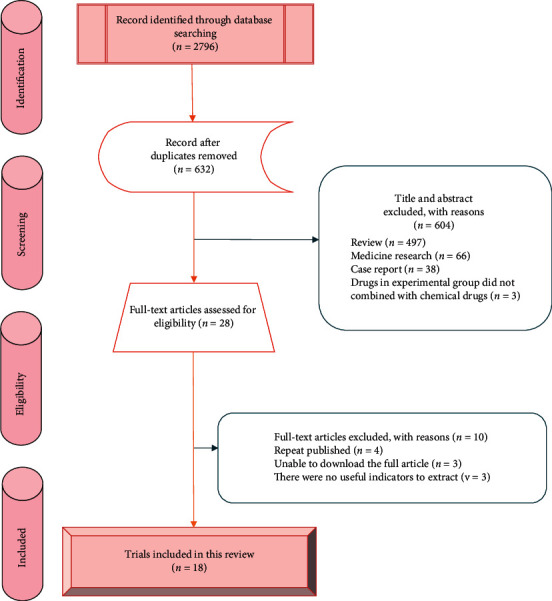
PRISMA flow diagram of literature selection.

Based on the above retrieval strategies, a total of 2796 potentially relevant trials were retrieved from 11 electronic databases, and 632 trials were retrieved after deleting 2164 duplicates. After reviewing the titles and abstracts, 604 articles were excluded because they did not comply with the inclusion criteria, and 28 trials initially met the predetermined requirements for detailed evaluation by reading the full text. Finally, 18 trials were included for meta-analysis [13–30]. The PRISMA flow diagram of the literature retrieval process was shown in Figure 1. All included trials have been published as full articles.

### 3.2. Basic Characteristics of the Included Studies

**Figure 2:**
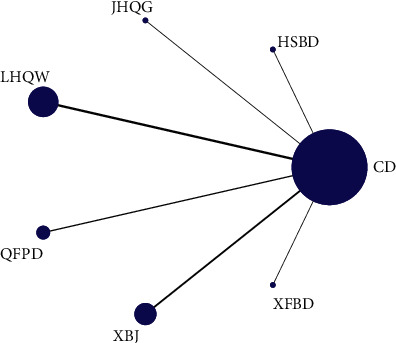
Network plot of the comparisons for the Bayesian network meta-analysis.

Table 1 summarized the basic characteristics of the eligible 18 trials, and a total of 2036 patients with COVID-19 were included. Sample sizes ranged from 12 to 295. In these 18 trials, 10 were randomized controlled trials [13, 15, 18, 19, 21, 23, 24, 27, 29, 30] and 8 were retrospective studies [14, 16, 17, 20, 22, 25, 26, 28]. 7 trials used LHQW *vs.* CD [14, 16, 18, 22, 25–27], 5 trials used XBJ *vs.* CD [13, 17, 21, 23, 29], 3 used QFPD *vs.* CD [19, 20, 28], 1 used JHQG *vs.* CD [15], 1 used XFBD *vs.* CD [24], and 1 used HSBD *vs.* CD [30].  Figure 2 provided the network plot of all the included trials.  Table A.1 showed the pooled hazard ratios of different drugs for outcomes. In addition, the specific percentage ranking of competing drugs was revealed in Table A.2, and the drug ranking histogram was shown in Figure A.1.

### 3.3. Risk of Bias Assessment of the Literature Included in the Study

The methodological quality of 10 randomized controlled trials was summarized in Table A.3, and the criteria in the Cochrane Handbook for Systematic Reviews of Interventions were used to assess the risk of bias in the study. Although randomization was announced in all 10 trials, 5 trials used random number table [15, 18, 21, 23, 27], 1 used the R 3.6.2 software [30], 1 used the method of flip a coin [24], and 3 did not report the method [13, 19, 29]. Moreover, only 1 trial reported allocation concealment [21], and all the trials did not report the blind method. The quality of 8 retrospective studies was assessed by NOS.  Table A.4 summarized the NOS scores of each study, all of which were of fair quality. Since fewer than 10 trials were included in each subgroup, publication bias could not be adequately analyzed.

### 3.4. Outcomes

#### 3.4.1. Clinical Effect

**Figure 3:**
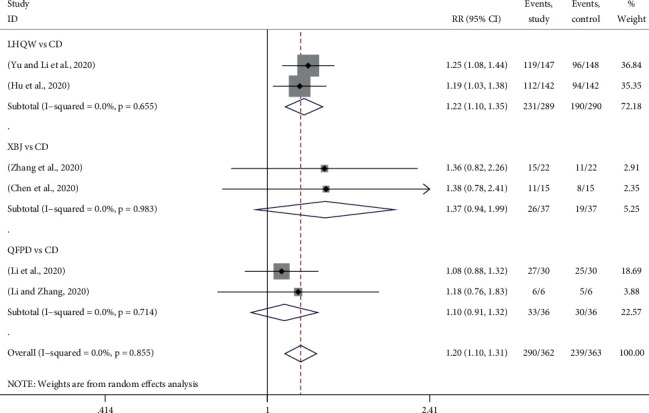
Forest plots for clinical effect by Bayesian network meta-analysis and traditional meta-analysis.

Clinical effect was reported in 6 trials, in which 2 used LHQW *vs.* CD [18, 27], 2 used XBJ *vs.* CD [13, 29], and 2 used QFPD *vs.* CD [19, 20].  Figure 3 provided the forest plots for the network meta-analysis of each relevant drug. The meta-analysis showed that using TMTP to treat COVID-19 could significantly increase the clinical effect (RR, 1.20; 95% CI, 1.10 to 1.31). Compared with CD, LHQW significantly improved the clinical efficacy, which was 1.22 times higher than that of CD (RR, 1.22; 95% CI, 1.10 to 1.35). However, there is no remarkable difference between XBJ and CD (RR, 1.37; 95% CI, 0.94 to 1.99) or QFPD and CD (RR, 1.10; 95% CI, 0.91 to 1.32) on clinical effect. It should be noted that the mixed treatment comparison and drug sequencing were not performed because there was no closed ring in the network plot.

#### 3.4.2. CT Recovery Rate

**Figure 4:**
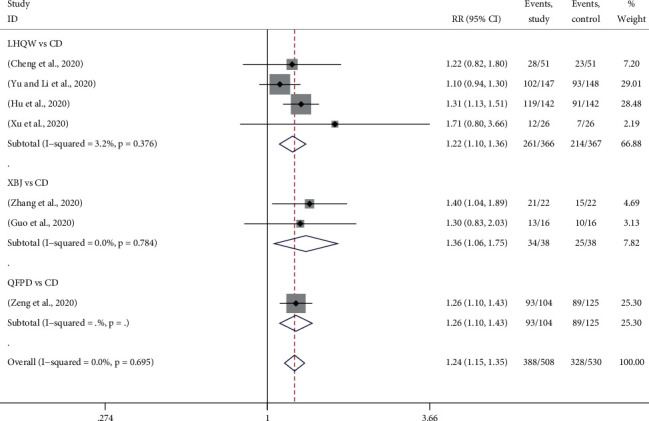
Forest plots for CT recovery rate by Bayesian network meta-analysis and traditional meta-analysis.

CT results are of great significance in the diagnosis of COVID-19, which is mainly characterized by ground-glass opacity. The improvement of COVID-19 was determined according to the CT changes before and after treatment in the clinic. A total of 7 trials reported the CT recovery of patients after treatment, in which 4 used LHQW *vs*. CD [14, 18, 25, 27], 2 used XBJ *vs*. CD [17, 29], and 1 used QFPD *vs*. CD [28].  Figure 4 provided the forest plots for the network meta-analysis of each relevant drug. The results exhibited that the CT recovery rate of TMTP was increased significantly more than that of CD alone (RR, 1.24; 95% CI, 1.15 to 1.35). The rates of LHQW, XBJ, and QFPD were 1.22 (RR, 1.22; 95% CI, 1.10 to 1.36), 1.36 (RR, 1.36; 95% CI, 1.06 to 1.75), and 1.26 (RR, 1.26; 95% CI, 1.10 to 1.43) times higher than those of CD. However, because there was no closed ring in the network plot, the mixed treatment comparison and drug sequencing were not performed.

#### 3.4.3. Nucleic Acid Negative Rate

2 trials reported the nucleic acid of patients before and after treatment [23, 29], all of which used XBJ *vs.* CD.  Figure A.2 showed the forest plots. However, compared with CD, XBJ did not show an obvious increasing efficacy on it (RR, 0.97; 95% CI, 0.72 to 1.29).

#### 3.4.4. Disappearance Rate of Primary Symptoms

**Figure 5:**
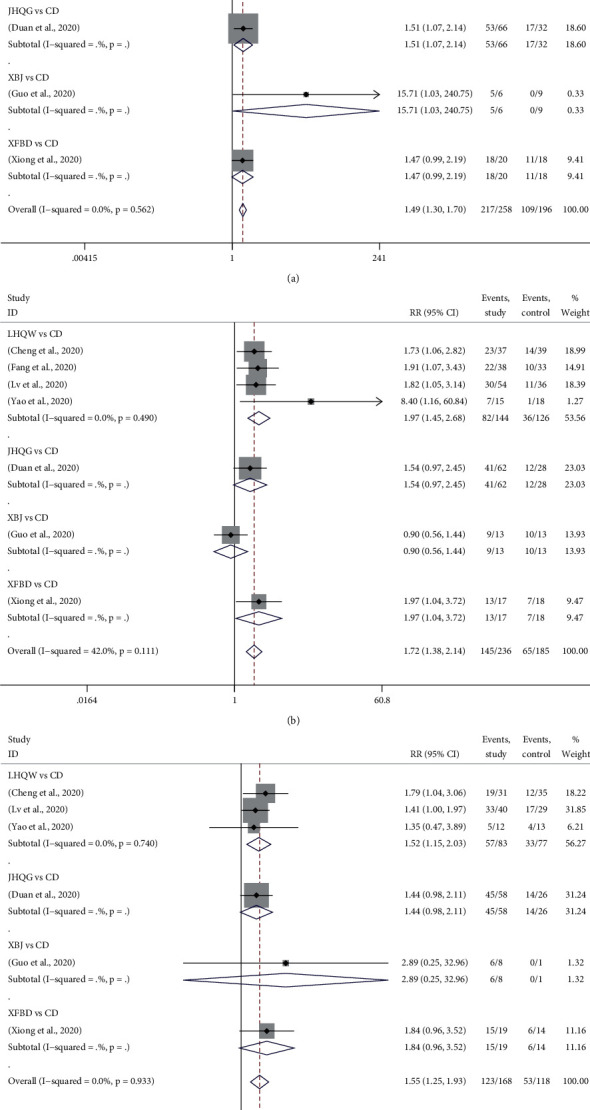
Forest plots for disappearance rate of primary symptoms by Bayesian network meta-analysis and traditional meta-analysis. (a) Fever. (b) cough. (c) fatigue.

The primary symptoms of COVID-19 patients were fever, cough, and fatigue. 7 trials reported the improvement of fever and cough after treatment [14–17, 22, 24, 26], and 6 reported fatigue [14, 15, 17, 22, 24, 26].  Figure 5 provided the forest plots for the network meta-analysis of each relevant drug. Compared with CD, the disappearance rates of fever (RR, 1.49; 95% CI, 1.30 to 1.70), cough (RR, 1.72; 95% CI, 1.38 to 2.14), and fatigue (RR, 1.55; 95% CI, 1.25 to 1.93) were greatly increased when using TMTP.

Compared with CD, LHQW showed a significant effect on increasing the disappearance rate of primary symptoms, which were 1.42 (RR, 1.42; 95% CI, 1.22 to 1.65), 1.97 (RR, 1.97; 95% CI, 1.45 to 2.68), and 1.52 (RR, 1.52; 95% CI, 1.15 to 2.03) times higher than those of CD in fever, cough, and fatigue respectively. JHQG and XBJ could remarkably improve the fever symptoms of patients, which were 1.51 (RR, 1.51; 95% CI, 1.07 to 2.41) and 15.71 (RR, 15.71; 95% CI, 1.03 to 240.75) times higher than those of CD, but they did not show an obvious advantage in cough and fatigue. A significant improvement of cough was observed by XFBD, whose efficacy was 1.97 times higher than that of CD (RR, 1.97; 95% CI, 1.04 to 3.72). However, there was no remarkable difference in fever and fatigue.

The results of network meta-analysis showed that XBJ was the most effective drug for improving fever, followed by XFBD, LHQW, JHQG, and CD. XFBD was the best drug for cough, followed by LHWE, JHQG, CD, and XBJ. In addition, XBJ was best for fatigue, followed by XFBD, LHQW, JHQG, and CD, while LHQW and JHQG had similar effects on fatigue.

#### 3.4.5. Disappearance Rate of Respiratory Symptoms

The respiratory symptoms of COVID-19 patients mainly included expectoration, shortness of breath, chest distress, rhinobyon, rhinorrhea, and sore throat. Expectoration was reported in 6 trials [14–17, 22, 26], and shortness of breath was reported in 5 trials additionally [14, 16, 22, 24, 26]; then, 4 trials reported chest distress [14, 22, 24, 26], rhinobyon [15, 16, 22, 26], rhinorrhea [15, 16, 22, 26], and sore throat [15, 22, 24, 26].  Figure A.3 provided the forest plots for the network meta-analysis of each relevant drug. Meta-analysis identified that TMTP could significantly improve expectoration (RR, 2.05; 95% CI, 1.28 to 3.30), shortness of breath (RR, 2.27; 95% CI, 1.33 to 3.86), and chest distress (RR, 2.24; 95% CI, 1.47 to 3.41) compared with CD, but it had no obvious advantages in rhinobyon, rhinorrhea, and sore throat.

Using LHQW to treat COVID-19 could obviously increase the disappearance rate of expectoration, shortness of breath, and chest distress, whose effects were 2.58 (RR, 2.58; 95% CI, 1.15 to 5.82), 2.79 (RR, 2.79; 95% CI, 1.29 to 6.02), and 2.15 (RR, 2.15; 95% CI, 1.38 to 3.33) times higher than those of CD. However, there was no remarkable difference between LHQW and CD on rhinobyon, rhinorrhea, and sore throat. JHQG could also improve expectoration, and its efficacy was 1.85 times higher than that of CD (RR, 1.85; 95% CI, 1.01 to 3.38), but it did not show good effects on rhinobyon, rhinorrhea, and sore throat. According to the results, there was no obvious difference between XFBD and CD in shortness of breath and chest distress, as well as QFBD and CD in sore throat.

The network meta-analysis exhibited that LHQW and JHQG were the best drugs for expectoration, followed by CD and XBJ. And LHQW was the most effective drug for shortness of breath, followed by XFBD and CD. XFBD was the best one in improving chest distress, followed by LHQW and CD. JHQG was the best drug for rhinobyon and rhinorrhea, in which the former was secondary to LHQW and CD, and the latter was secondary to CD and LHQW. Additionally, LHQW was the most effective for sore throat, followed by JHQG, CD, and XFBD.

#### 3.4.6. Disappearance Rate of Gastrointestinal Symptoms

The gastrointestinal symptoms of COVID-19 patients mainly included nausea, diarrhea, emesis, and inappetence. 5 trials reported nausea [14, 15, 22, 24, 26] and diarrhea [15, 17, 22, 24, 26], and 4 reported emesis [15, 22, 24, 26] and inappetence [14, 22, 24, 26].  Figure A.4 provided the forest plots for the network meta-analysis of each relevant drug. However, the meta-analysis evaluated that there was no significant difference between TMTP and CD in the disappearance rate of nausea, diarrhea, emesis, and inappetence. LHQW and XFBD did not show a remarkable advantage on them, just as JHQG on nausea, diarrhea, and emesis and XBJ on diarrhea.

The results of the network meta-analysis identified that LHQW was the most effective in nausea, followed by JHQG, CD, and XFBD. XBJ was the best drug in improving diarrhea, followed by LHQW, CD, XFBD, and JHQG. For emesis, the drug sorts were LHQW, JHQG, CD, and XFBD. And LHQW was also the best drug for inappetence, followed by XFBD and CD.

#### 3.4.7. Disappearance Rate of Other Symptoms

5 trials reported muscle soreness before and after treatment [14–16, 22, 26], and 3 reported headache [22, 24, 26].  Figure A.5 provided the forest plots for the network meta-analysis of each relevant drug. Compared with CD, TMTP exhibited significant effects on increasing the disappearance rate of muscle soreness (RR, 1.51; 95% CI, 1.03 to 2.20), but there was no obvious difference between TMTP and CD on headache. 4 trials comparing LHQW with CD reported muscle soreness before and after the intervention. The meta-analysis revealed that LHQW significantly improved its efficacy, which was 1.88 times higher than that of CD (RR, 1.88; 95% CI, 1.05 to 3.36); however, it did not show a remarkable advantage on headache. Either JHQG or XFBD was not more effective than CD in the treatment of muscle soreness and headache.

According to the results of network meta-analysis, LHQW was the best drug for improving muscle soreness and headache, followed by JHQG and CD for muscle soreness, and XFBD and CD for headache.

#### 3.4.8. Inflammatory Biomarkers (CRP and WBC)

4 trials reported CRP level before and after treatment [13, 17, 23, 27] and 3 reported WBC [17, 20, 23].  Figure A.6 provided the forest plots for the network meta-analysis of each relevant drug. Meta-analysis showed that using TMTP could significantly decrease CRP level (MD, −0.94; 95% CI, −1.79 to −0.09); however, there was no obvious difference between TMTP and CD in WBC. The reduction in CRP was remarkably greater for XFBD than that of CD (MD, −0.46; 95% CI, −0.69 to −0.23). But XBJ had no obvious advantages in decreasing the levels of CRP and WBC, and there was no significant difference between QFPD and CD in WBC.

The network meta-analysis results identified that QFPD was the best drug for decreasing CRP levels, followed by CD and XBJ, and XBJ was the best for WBC, followed by CD and QFPD.

#### 3.4.9. Exacerbation Rate

**Figure 6:**
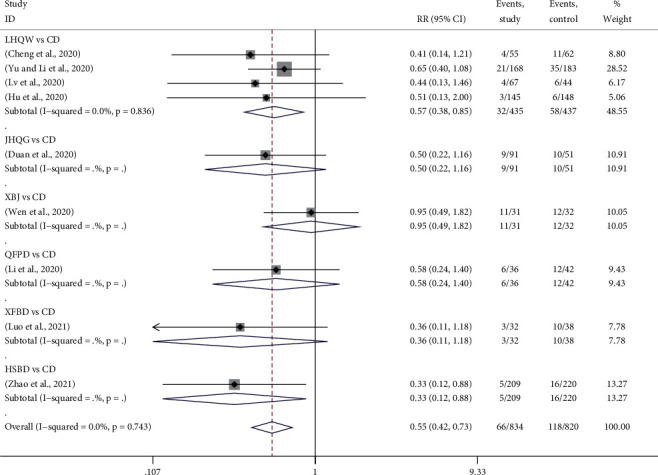
Forest plots for exacerbation rate by Bayesian network meta-analysis and traditional meta-analysis.

Exacerbation after treatment was reported in 9 trials, in which 4 used LHQW *vs.* CD [14, 18, 22, 27], 2 used XBJ *vs.* CD [21, 23], 1 used JHQG *vs.* CD [15], 1 used QFPD *vs.* CD [20], and 1 used HSBD *vs.* CD [30].  Figure 6 provided the forest plots for the network meta-analysis of each relevant drug. Meta-analysis exhibited that using TMTP to treat COVID-19 could significantly reduce the exacerbation rate of patients (RR, 0.55; 95% CI, 0.42 to 0.73). Compared with CD, exacerbation rates of LHQW and HSBD groups were significantly reduced, which were 0.57 (RR, 0.57; 95% CI, 0.38 to 0.85) and 0.33 (RR, 0.33; 95% CI, 0.012 to 0.88) times than those of CD. However, there was no obvious difference between JHQG, QFPD, XBJ, or XFBD and CD.

The network meta-analysis evaluated that using XFBD to treat COVID-19 had the least exacerbation number, followed by HSBD, JHQG, LHQW, QFPD, XBJ, and CD.

#### 3.4.10. Adverse Reactions

**Figure 7:**
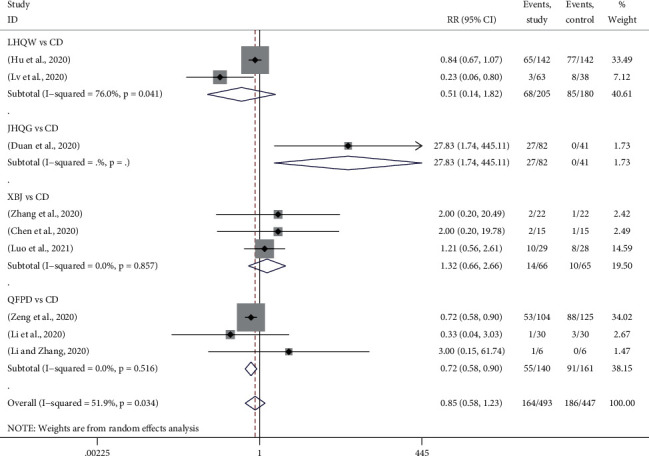
Forest plots for disappearance rate of adverse reaction by Bayesian network meta-analysis and traditional meta-analysis.

Adverse reactions were reported in 9 trials, in which 3 trials used QFPD *vs.* CD [19, 20, 28], 3 used XBJ *vs.* CD [13, 21, 29], 2 used LHQW *vs.* CD [18, 22], and 1 used JHQG *vs.* CD [15].  Figure 7 showed the forest plots for the network meta-analysis of each relevant drug. Compared with CD, the rate of adverse reaction in the QFPD group was significantly lower, which was 0.72 times that of CD (RR, 0.72; 95% CI, 0.58 to 0.90). However, LHQW and XBJ did not show good advantages in lowering adverse reactions.

The results of network meta-analysis showed that using LHQW to treat COVID-19 could produce the least adverse reaction, followed by QFPD, CD, XBJ, and JHQG.

## 4. Discussion

The efficacy and safety of TMTP for COVID-19 were evaluated by Bayesian network meta-analysis. 18 trials that contained 2036 participants were included. The traditional meta-analysis exhibited that using LHQW to treat COVID-19 could significantly increase the efficacy, and its clinical and CT effect was 1.22 times higher than that of CD, which could also improve most of the symptoms. Its effects on fever, cough, fatigue, expectoration, shortness of breath, chest distress, and muscle soreness were 1.42, 1.97, 1.52, 2.58, 2.79, 2.15, and 1.88 times those of CD, respectively, and the exacerbation rate was 0.52 times that of CD. The CT effects of XBJ and QFPD were 1.36 and 1.26 times those of CD, and the adverse reaction rate of QFPD was 0.72 times that of CD. The effects of JHQG on the improvement of fever and expectoration were 1.51 and 1.85 times those of CD. The cough effect and exacerbation rate of XFBD were 1.97 and 0.29 times those of CD, and it could also lower than the CRP level. The network meta-analysis identified that LHQW was the most effective drug in improving expectoration, shortness of breath, sore throat, nausea, emesis, inappetence, muscle soreness, and headache of COVID-19 patients, and it could produce the least adverse reactions. XBJ was the most effective in improving fever, fatigue, and diarrhea, and it showed great advantages in lowering WBC levels. XFBD was the best drug in improving cough and chest distress, and it had the least exacerbation rate. JHQG was the best one in improving rhinobyon and rhinorrhea, and QFPD was the most effective drug in decreasing CRP levels.

Some clinical studies have proved the efficacy and safety of TCM in the treatment of COVID-19. It was shown that TCM treatment of COVID-19 could significantly reduce the mortality of patients and delay the progression of the disease, especially in the treatment of severe/critical cases, which showed good advantages [31]. Furthermore, it could significantly improve clinical remission rates and shorten the nucleic acid conversion time and hospitalization time. The combination of HSBD and TCM injection showed obvious superiority in the treatment of COVID-19 [32]. Meanwhile, HSBD alone could also remarkably shorten the fever time of COVID-19 patients, relieve symptoms such as cough, fatigue, and chest discomfort, and improve the CT recovery rate [33]. Based on the two indicators of exacerbation rate and adverse reactions, this study showed that TCM could effectively prevent the deterioration and progression of the disease and had good safety. It could not only obviously relieve the multisystem clinical symptoms of COVID-19 patients and reduce clinical indicators but also significantly improve the clinical efficacy, which is a powerful and effective measure for the treatment of COVID-19.

Each prescription in TMTP is a combination of classical and famous formulae, which can play a role in the treatment of COVID-19 through multitarget comprehensive intervention. They can be used clinically in combination with the actual situation of patients and are suitable for the treatment of mild, common, and severe COVID-19 patients.

LHQW is a combination of *Maxing Shigan Decoction* and *Yinqiao Powder*, which is composed of 12 kinds of herbs including Lianqiao (fructus of *Forsythia suspensa* (Thunb.) Vahl), Jinyinhua (floral bud of *Lonicera japonica* Thunb), Mahuang (herb of *Ephedra equisetina* Bge.), Xingren (seed of *Prunus armeniaca* L. var. *ansu* Maxim), Banlangen (root of *Isatis indigotica* Fort), Guanzhong (rhizome of *Dryopteris crassirhizoma* Nakai), Yuxingcao (herb of *Houttuynia cordata* Thunb.), Huoxiang (wrinkled herb of *Agastache rugosus* (Fisch. et Mey. O. Ktze.), Dahuang (radix and rhizome of *Rheum palmatum* L.), Hongjingtian (radix and rhizome of *Rhodiola rosea* L.), Bohe (herb of *Mentha haplocalyx* Briq.), Gancao (radix and rhizome of *Glycyrrhiza uralensis* Fisch.), and a mineral drug Shigao (*Gypsum fibrosum*). Lianqiao and Jinyinhua clear heat-toxicity and dispel wind pathogens; Mahuang disperses lung qi and dissipates phlegm; Shigao clears heat; Xingren improves cough and asthma; Banlangen cools blood to relieve sore throat; Guanzhong and Yuxingxao remove toxicity for eliminating carbuncles; Bohe dispels wind pathogens and relieves sore throat; Huoxiang removes dampness for regulating stomach; Dahuang eliminates heat; Hongjingtian moistens lung for arresting cough; Gancao clears heat-toxicity. Thus, LHQW has the effects of dispelling disease and detoxification, as well as relieving heat in the lung. Studies have shown that LHQW could significantly inhibit the replication of SARS-CoV-2 in Vero E6 cells at the mRNA level and greatly reduce the production of proinflammatory cytokines TNF-*α*, IL-6, CCL-2/MCP-1, and CXCL-10/IP-10, thus playing a role in the resistance to the virus; furthermore, it has a broad-spectrum effect on a series of influenza viruses by inhibiting virus proliferation and regulating immune function [34], which can not only enhance the body's immunity and inhibit respiratory inflammation [35] but also affect the relevant cytokines and ameliorate lung injury associated with inflammatory cell infiltration [36]. Therefore, LHQW has antibacterial, antipyretic, analgesic, anti-inflammatory, cough relieving, and expectorant effects [37], which can significantly improve flu-like symptoms such as phlegm, shortness of breath, sore throat, headache, muscle soreness, and inappetite.

JHQG integrates the classic formulae *Maxing Shigan Decoction*, *Yinqiao Powder,* and *Baihu Decoction* into one, which has the effects of dispelling wind and heat pathogens and clearing heat toxicity. It includes Lianqiao (fructus of *Forsythia suspensa* (Thunb.) Vahl), Jinyinhua (floral bud of *Lonicera japonica* Thunb), Mahuang (herb of *Ephedra equisetina* Bge.), Xingren (seed of *Prunus armeniaca* L. var. *ansu* Maxim), a mineral drug Shigao (*Gypsum fibrosum*), Huangqin (radix of *Scutellaria baicalensis* Georgi), Zhebeimu (bulb of *Fritillaria thunbergii* Miq.), Zhimu (rhizome of *Anemarrhena asphodeloides* Bge.), Niubangzi (fructus of *Arctium lappa* L.), Qinghao (herb of *Artemisia annua* L.), Bohe (herb of *Mentha haplocalyx* Briq.), and Gancao (radix and rhizome of *Glycyrrhiza uralensis* Fisch.). Lianqiao and Jinyinhua clear heat-toxicity and dispel wind pathogens; Mahuang disperses lung qi and dissipates phlegm; Shigao clears heat; Xingren improves cough and asthma; Huangqin dispels heat and removes dampness; Zhebeimu reduces phlegm; Zhimu nourishes yin and clears heat; Niubangzi dispels heat pathogens and relieves throat disorder; Qinghao and Gancao clear heat-toxicity; Bohe dispels wind pathogens and relieves sore throat. JHQG could inhibit the replication of the influenza virus, promote virus clearance [38], alleviate rhinobyon, rhinorrhea, and other symptoms through a variety of mechanisms, and shorten the fever time by PTGS2 possibly [39].

XBJ is developed on the basis of *Xuefu Zhuyu Decoction*, including Honghua (floral bud of *Carthamus tinctorius* L.), Chishao (radix of *Paeonia lactiflora* Pall.), Chuanxiong (rhizome of *Ligusticum chuanxiong* Hort.), Danshen (radix and rhizome of *Salvia miltiorrhiza* Bge.), and Danggui (radix of *Angelica sinensis* (Oliv.) Diels), which is used for the mutual syndrome of stasis and poison in warm and hot diseases. XBJ has the function of activating blood circulation and removing blood stasis, which can antagonize endotoxin *in vitro*. It can not only improve a variety of infectious symptoms such as fever and fatigue by anti-inflammatory, antiendotoxin, and the improvement of blood coagulation function and alleviate digestive symptoms such as abdominal pain and diarrhea by reducing the inflammatory response of the body but also shorten the recovery time of white blood cells [40]. Furthermore, it could effectively reduce acute lung injury by regulating the expression of pulmonary inflammatory factors p-p38 MAPK, NF-*κ*B 65, HIF-1*α*, p-I*κ*B-*α,* and TGF-*β*1 [41] and improve dyspnea and hypoxemia in patients with severe COVID-19.

QFPD integrates *Maxing Shigan Decoction*, *Shegan Mahuang Decoction*, *Xiao Chaihu Decoction*, and *Wuling Powder*, which includes Mahuang (herb of *Ephedra equisetina* Bge.), Xingren (seed of *Prunus armeniaca* L. var. *ansu* Maxim), a mineral drug Shigao (*Gypsum fibrosum*), Guizhi (twig of *Cinnamomum cassia* Presl), Zexie (bulb of *Alisma orientate* (Sam.) Juzep), Zhuling (sclerotium of *Polyporus umbellatus* (Pers.) Frie), Chaihu (radix of *Bupleurum chinense* DC.), Huangqin (radix of *Scutellaria baicalensis* Georgi), Banxia (tuber of *Pineilia ternata* (Thunb.) Breit), Shengjiang (rhizome of *Zingiber officinale* Rose), Ziyuan (rhizome of *Aster tataricus* L. f.), Kuandonghua (floral bud of *Tussilago farfara* L.), Shegan (rhizome of *Belamcanda chinensis* (L.) DC.), Xixin (radix and rhizome of *Asarum heterotropoides* Fr. Schmidt var. *mandshuricum* (Maxim) Kitag.), Shanyao (rhizome of *Dioscorea opposita* Thunb.), Zhishi (fructus of *Citrus aurantium* L.), Chenpi (pericarp of *Citrus reticulata* Blanco), Huoxiang (wrinkled herb of *Agastache rugosus* (Fisch. et Mey. O. Ktze.), and Gancao (radix and rhizome of *Glycyrrhiza uralensis* Fisch.). QFPD has effects of clearing heat toxicity, dispersing lung qi, and dissipating phlegm. It could inhibit proinflammatory cytokines such as IL-6 and IL-1*β*, increase anti-inflammatory cytokines such as IL-4 and IL-10, or inhibit NF-*κ*B and MAPK signaling pathways, exert an anti-inflammatory and antiviral role, and significantly reduce inflammatory indicators [42, 43].

XFBD is a combination of *Maxingshigan Decoction*, *Maxingyigan Decoction*, *Tinglidazaoxiefei Decoction,* and *Qianjin Weijing Decoction*, including Mahuang (herb of *Ephedra equisetina* Bge.), Xingren (seed of *Prunus armeniaca* L. var. *ansu* Maxim), a mineral drug Shigao (*Gypsum fibrosum*), Yiyiren (seed of *Coix lacryma-jobi* L. *var. ma-yuen* (Roman.) Stapf), Qinghao (herb of *Artemisia annua* L.), Huzhang (rhizome and radix of *Polygonum cuspidatum* Sieb. et Zucc), Mabiancao (herb of *Verbena officinalis* L.), Lugen (rhizome of *Phragmites communis* Trin), Tinglizi (seed of *Descurainia sophia* (L.) Webb. ex Prantl.), Juhong (pericarp of *Citrus reticulata* Blanco), and Gancao (radix and rhizome of *Glycyrrhiza uralensis* Fisch.). XFBD has the effects of clearing heat toxicity, dispersing lung qi, and dissipating phlegm. It may block inflammatory cytokine storm by inhibiting excessive cytokine production, immune cell activation, and oxidative damage *in vivo*, which is the intervention mechanism after virus invasion [44]. Therefore, XFBD can significantly relieve cough, chest tightness, and other symptoms of patients and inhibit the progression of the disease.

HSBD integrates *Maxingshigan Decoction* and *Tinglidazaoxiefei Decoction* into one, including Mahuang (herb of *Ephedra equisetina* Bge.), Xingren (seed of *Prunus armeniaca* L. var. *ansu* Maxim), a mineral drug Shigao (*Gypsum fibrosum*), Huoxiang (wrinkled herb of *Agastache rugosus* (Fisch. et Mey. O. Ktze.), Houpo (velamen of *Magnolia officinalis* Rehd. et Wils.), Cangzhu (rhizome of *Atractylodes lancea* (Thunb.) DC.), Caoguo (fructus of *Amomum tsao-ko* Crevost et Lemaire), Banxia (tuber of *Pineilia ternata* (Thunb.) Breit), Fuling (sclerotium of *Poria cocos* (Schw) Wolf), Dahuang (radix and rhizome of *Rheum palmatum* L.), Yujin (earthnut of *Curcuma xvenyujin* Y. H. Chen et C. Ling), Huangqi (root of *Astragalus membranaceus* (Fisch.) Bge. *var. mongholicus* (Bge.) Hsiao), Tinglizi (seed of *Descurainia sophia* (L.) Webb. ex Prantl.), Chishao (radix of *Paeonia lactiflora* Pall.), and Gancao (radix and rhizome of *Glycyrrhiza uralensis* Fisch.). HSBD has the effects of dispelling lung heat, preventing asthma, drying damp, strengthening the spleen, and removing blood stasis. It could inhibit the infection, replication, and proliferation of the SARS-CoV-2 virus to some extent, regulate the balance of the RAS system and inflammatory response accordingly, and effectively block the formation of the inflammatory storm after the infection of the SARS-CoV-2 virus [45, 46].

There were some important advantages in this study. Methodologically, our study benefits from rigorous methods, extensive search, repeated and independent screening, meticulous data abstraction process, and comprehensiveness of analytical indicators. In addition, the Bayesian network meta-analysis was used to compare therapies indirectly when no head-to-head trial existed, and more accurate evaluation for efficacy was obtained by jointly assessing direct and indirect comparisons. Moreover, we mapped drug sequencing figures through single and mixed analysis, further ranked the competing drugs, and summed up the best one for that outcome.

The following limitations should be considered in this study. Due to the insufficient sample size, short duration, and partial retrospective study of TMTP in the treatment of COVID-19, the methodological quality had a certain risk bias. In addition, TMTP is mainly used in China because of the limited application of TCM in other countries. The Chinese government has issued health packages to Chinese people all over the world, while the data cannot be calculated and summarized well. Therefore, although TMTP is widely used, the data is limited, which will have a certain impact on the results.

## 5. Conclusion

In summary, the study focused on the diversity of symptoms in COVID-19 patients, which was the most large-scale and comprehensive research on this issue so far. In this study, multiple outcomes were used to systematically evaluate TMTP efficacy for COVID-19 so as to determine the precise efficacy and safety of each drug for COVID-19 treatment. Bayesian network meta-analysis was used to integrate clinical evidence from direct and indirect treatment comparisons into a network, using a common comparator for indirect comparisons in the absence of head-to-head trials, and the competing drugs in each outcome were ranked. LHQW could significantly reduce proinflammatory cytokines and enhance immunity with antiviral effects. JHQG could inhibit influenza virus replication and promote virus clearance. XBJ could control infectious symptoms and alleviate acute lung injury effectively. QFPD had anti-inflammatory and antiviral effects, which could significantly reduce inflammatory indicators. XFBD could block the inflammatory cytokine storm and intervene in virus invasion. And HSBD could inhibit the infection, replication, and proliferation of the SARS-CoV-2 virus to a certain extent and, obviously, block the formation of the inflammatory storm after infection. As a result, LHQW could improve most symptoms of COVID-19 patients with no obvious adverse reactions. Therefore, we believe that it should be firstly recommended for COVID-19 treatment, especially for critically ill patients. Patients with severe fever, fatigue, and diarrhea could be treated by XBJ, while patients with obvious rhinobyon and rhinorrhea could choose JHQG. XFBD is recommended for patients with cough and chest tightness as the main manifestation, and HSBD could significantly lower the exacerbation rate. Our findings will help to provide guidance for COVID-19 treatment and future research.

## Figures and Tables

**Table 1:** Characteristics of the 18 trials included in the Bayesian network meta-analysis.

Author(s)	Sample size (experimental/control)	Time frame (y)	Contrast drugs	Duration	Outcome measures
Chen et al. (2020)	15/15	2020.1–2020.3	XBJ *vs.* CD	14 days	Clinical effect, CRP, adverse reactions.
Cheng et al. (2020)	51/51	2020.1.1–2020.1.30	LHQW *vs.* CD	7 days	CT, fever, cough, fatigue, expectoration, shortness of breath, chest distress, nausea, inappetence, muscle soreness, exacerbation rate.
Duan et al. (2020)	82/41	2020.2.1–2020.2.5	JHQG *vs.* CD	5 days	Fever, cough, fatigue, expectoration, rhinobyon, rhinorrhea, sore throat, nausea, diarrhea, emesis, muscle soreness, exacerbation rate, adverse reactions.
Fang et al. (2020)	42/41	2020.1.28–2020.3.31	LHQW *vs.* CD	NR	Fever, cough, expectoration, shortness of breath, rhinobyon, rhinorrhea, muscle soreness.
Guo et al. (2020)	16/16	2020.1.20–2020.3.11	XBJ *vs.* CD	NR	CT, fever, cough, fatigue, expectoration, diarrhea, CRP, WBC.
Hu et al. (2020)	142/142	2020.2.2–2020.2.15	LHQW *vs.* CD	14 days	Clinical effect, CT, exacerbation rate, adverse reactions.
Li and Zhang (2020)	6/6	2020.2–2020.3	QFPD *vs.* CD	6 days	Clinical effect, WBC, adverse reactions.
Li et al. (2020)	30/30	2020.1.24–2020.3.7	QFPD *vs.* CD	NR	Clinical effect, exacerbation rate, adverse reactions.
Luo et al. (2021)	29/28	2020.2.16–2020.3.25	XBJ *vs.* CD	14 days	Exacerbation rate, adverse reactions.
Lv et al. (2020)	63/38	2020.1.1–2020.1.27	LHQW *vs.* CD	10 days	Fever, cough, fatigue, expectoration, shortness of breath, chest distress, rhinobyon, rhinorrhea, sore throat, nausea, diarrhea, emesis, inappetence, muscle soreness, headache, exacerbation rate, adverse reactions.
Wen et al. (2020)	20/20	2020.1–2020.3	XBJ *vs.* CD	7 days	NANR, CRP, WBC, exacerbation rate.
Xiong et al. (2020)	22/20	2020.1.30–2020.2.10	XFBD *vs.* CD	7 days	Fever, cough, fatigue, shortness of breath, chest distress, sore throat, nausea, diarrhea, emesis, inappetence, headache.
Xu et al. (2020)	26/26	2020.1.29–2020.2.28	LHQW *vs.* CD	7 days	CT.
Yao et al. (2020)	21/21	2020.1.11–1.30	LHQW *vs.* CD	NR	Fever, cough, fatigue, expectoration, shortness of breath, chest distress, rhinobyon, rhinorrhea, sore throat, nausea, diarrhea, emesis, inappetence, muscle soreness, headache.
Yu et al. (2020)	147/148	2020.2.17–2020.3.6	LHQW *vs.* CD	7 days	Clinical effect, CT, CRP, exacerbation rate.
Zeng et al. (2020)	104/125	2019.12–2020.3	QFPD *vs.* CD	NR	CT, adverse reactions.
Zhang et al. (2020)	22/22	2020.1.21–2020.2.24	XBJ *vs.* CD	7 days	Clinical effect, CT, NANR, adverse reactions.
Zhao et al. (2021)	204/204	2020.2.13–3.7	HSBD *vs.* CD	7 days	Exacerbation rate.

Abbreviations: CD, chemical drugs; CRP, C-reactive protein; HSBD, *Hua-Shi-Bai-Du;* JHQG, *Jin-Hua-Qing-Gan*; LHQW, *Lian-Hua-Qing-Wen*; NANR, nucleic acid negative rate; NR, not reported; QDPD, *Qing-Fei-Pai-Du*; WBC, white blood cell; XBJ, *Xue-Bi-Jing*; XFBD, *Xuan-Fei- Bai-Du*.

## Data Availability

The data used to support the findings of this study are available from the corresponding author upon request.

## Abbreviations

CD: Chemical drugs
CI: Confidence interval
COVID-19: Coronavirus disease 2019
CRP: C-reactive protein
JHQG: *Jin-Hua-Qing-Gan*
HSBD: *Hua-Shi-Bai-Du*
LHQW: *Lian-Hua-Qing-Wen*
MD: Mean difference
NOS: Newcastle Ottawa scale
QFPD: *Qing-Fei-Pai-Du*
RR: Relative risk
TCM: Traditional Chinese medicine
TMTP: Three Chinese patent medicines and three TCM prescriptions
WBC: White blood cell
WHO: World Health Organization
XBJ: *Xue-Bi-Jing*
XFBD: *Xuan-Fei-Bai-Du*.

## Supplementary Materials

Supplementary Materials Supplementary materials provide the methodology quality of included trials and competitive drug sequencing in the study. Forest plots for nucleic acid negative rate and the disappearance rate of respiratory, gastrointestinal, and other symptoms as well as inflammatory biomarkers are also shown. They include pooled hazard ratios for competing drugs in each outcome (Table A1), specific percentage ranking in terms of competing drugs in each outcome (Table A2), methodology quality of the 10 included randomized controlled trials according to the Cochrane handbook (Table A3), rank probability of competing drugs in each outcome (Figure A1), forest plots for nucleic acid negative rate by Bayesian network meta-analysis and traditional meta-analysis (Figure A2), forest plots for disappearance rate of respiratory symptoms by Bayesian network meta-analysis and traditional meta-analysis (Figure A3), forest plots for disappearance rate of gastrointestinal symptoms by Bayesian network meta-analysis and traditional meta-analysis (Figure A4), forest plots for disappearance rate of other symptoms by Bayesian network meta-analysis and traditional meta-analysis (Figure A5), and forest plots for disappearance rate of inflammatory biomarkers by Bayesian network meta-analysis and traditional meta-analysis (Figure A6).

## References

[1] Rothan H. A., Byrareddy S. N. (2020). The epidemiology and pathogenesis of coronavirus disease (COVID-19) outbreak. *Journal of Autoimmunity*.

[2] Wang C., Horby P. W., Hayden F. G., Gao G. F. (2020). A novel coronavirus outbreak of global health concern. *The Lancet*.

[3] Wissem H., Nadia B. L. (2020). COVID-19: main therapeutic options. *Tunisie Medicale*.

[4] Ren J.-l., Zhang A.-H., Wang X.-J. (2020). Traditional Chinese medicine for COVID-19 treatment. *Pharmacological Research*.

[5] Catalá-López F., Tobías A., Cameron C., Moher D., Hutton B. (2014). Network meta-analysis for comparing treatment effects of multiple interventions: an introduction. *Rheumatology International*.

[6] Ades A. E., Sculpher M., Sutton A. (2006). Bayesian methods for evidence synthesis in cost-effectiveness analysis. *PharmacoEconomics*.

[7] Song F., Altman D. G., Glenny A. M., Deeks J. J. (2003). Validity of indirect comparison for estimating efficacy of competing interventions: empirical evidence from published meta-analyses. *BMJ*.

[8] Sutton A., Ades A. E., Cooper N., Abrams K. (2008). Use of indirect and mixed treatment comparisons for technology assessment. *PharmacoEconomics*.

[9] Moher D., Liberati A., Tetzlaff J., Altman D. G. (2009). Preferred reporting items for systematic reviews and meta-analyses: the PRISMA statement. *PLoS Medicine*.

[10] National Health Commission of the People’s Republic of China (2020). *Guidelines for the Diagnosis and Treatment of COVID-19 Pneumonia*.

[11] Higgins J. P. T., Green S. (2014). *Cochrane Reviewers’ Handbook 5.3.0*.

[12] Sedgwick P., Marston L. (2015). How to read a funnel plot in a meta-analysis. *BMJ*.

[13] Chen L. Z., Liu H., Xiao G. L. (2020). Therapeutic effect of Xuebijing injection in COVID-19 and its effect on CRP. *Journal of Chiniese Prescription Drug.*.

[14] Cheng D. Z., Wang W. J., Li Y., Wu X. D., Zhou B., Song Q. Y. (2020). Analysis of curative effect of 51 patients with novel coronavirus pneumonia treated with Chinese medicine Lianhua Qingwen: a multicentre retrospective study. *Tianjin Journal of Traditional Chinese Medicine*.

[15] Duan C., Xia G. W., Zheng C. J. (2020). Clinical observation on Jinhua Qinggan Granule combined with conventional western medicine therapy in treating mild cases of coronavirus disease 2019. *Journal of Traditional Chinese Medicine*.

[16] Fang F., Yang L., Qin S. C., Jiao R. (2020). Lianhua Qingwen granule in the treatment of 42 children suspected cases of COVID-19. *Chinese Journal of New Drugs*.

[17] Guo H., Zheng J., Xiang G. (2020). Xuebijing injection in the treatment of COVID-19: a retrospective case-control study. *Annals of Palliative Medicine*.

[18] Hu K., Guan W. J., Bi Y. (2020). Efficacy and safety of Lianhuaqingwen capsules, a repurposed Chinese herb, in patients with coronavirus disease 2019: a multicenter, prospective, randomized controlled trial. *Phytomedicine*.

[19] Li Y. D., Zhang W. J. (2020). Evaluation on the clinical effect of traditional Chinese medicine and western medicine regimens on COVID-19. *Guangming Journal of the Chinese Medical Association*.

[20] Li K. Y., An W., Xia F. (2020). Observation on clinical effect of modified Qingfei Paidu Decoction in treatment of COVID-19. *Chinese Traditional and Herbal Drugs*.

[21] Luo Z., Chen W., Xiang M. (2021). The preventive effect of Xuebijing injection against cytokine storm for severe patients with COVID-19: a prospective randomized controlled trial. *European Journal of Integrative Medicine*.

[22] Lv R. B., Wang W. J., Li X. (2020). Clinical observation on Lianhua Qingwen granules combined with western medicine conventional therapy in the treatment of 63 suspected cases of coronavirus disease 2019. *Journal of Traditional Chinese Medicine*.

[23] Wen L., Zhou Z. J., Jiang D. X., Huang K. (2020). Effect of xuebijing injection on inflammatory indicators and outcome of patients with severe COVID-19. *Chinese Critical Care Medicine*.

[24] Xiong W.-z., Wang G., Du J., Ai W. (2020). Efficacy of herbal medicine (Xuanfei Baidu decoction) combined with conventional drug in treating COVID-19:A pilot randomized clinical trial. *Integrative Medicine Research*.

[25] Xu X. H., Dong H., Tu S. H. (2020). Retrospective analysis of Jinye Budu granule and Lianhua Qinwen capsule in the treatment of common type COVID-19. *Res. Integrated Traditional and Western Medicine*.

[26] Yao K. T., Liu M. Y., Li X., Huang J. H., Cai H. B. (2020). Retrospective clinical analysis on treatment of coronavirus disease 2019 with traditional Chinese medicine Lianhua Qingwen. *Chin. Exp. Tradit. Med. Form.*.

[27] Yu P., Li Y. Z., Wan S. B., Wang Y. (2020). Effects of Lianhua Qingwen granules plus arbidol on treatment of mild corona virus disease-19. *Chinese Pharmaceutical Journal*.

[28] Zeng X. H., Ma W. H., Wang J. (2020). Effect of Qingfei Paidu decoction on clinical efficacy of COVID-19 pneumonia with Phlegm heat blocking lung. *West China Medical Journal,*.

[29] Zhang C. Y., Li Z. H., Zhang S., Wang W., Jiang X. Q. (2020). Clinical observation of Xuebijing in the treatment of COVID-19. *Chinese Journal of Hospital Pharmacy*.

[30] Zhao C., Li L., Yang W. (2021). Chinese medicine formula Huashibaidu granule early treatment for mild COVID-19 patients: an unblinded, cluster-randomized clinical trial. *Frontiers in Medicine*.

[31] Shu Z., Chang K., Zhou Y. (2021). Add-on Chinese medicine for coronavirus disease 2019 (ACCORD): a retrospective cohort study of hospital registries. *The American Journal of Chinese Medicine*.

[32] Shi N., Guo L., Liu B. (2021). Efficacy and safety of Chinese herbal medicine versus Lopinavir-Ritonavir in adult patients with coronavirus disease 2019: a non-randomized controlled trial. *Phytomedicine*.

[33] Liu J., Yang W., Liu Y. (2021). Combination of Hua Shi Bai Du granule (Q-14) and standard care in the treatment of patients with coronavirus disease 2019 (COVID-19): a single-center, open-label, randomized controlled trial. *Phytomedicine*.

[34] Li R. F., Hou Y. L., Huang J. C. (2020). Lianhuaqingwen exerts anti-viral and anti-inflammatory activity against novel coronavirus (SARS-CoV-2). *Pharmacological Research*.

[35] Wang S. H., Liu J. F., Zhang Y. L., Dong Z. (2019). Systematic review of efficacy and safety of lianhua qingwen capsules in treatment of viral influenza. *China Journal of Chinese Materia Medica*.

[36] Ding Y., Zeng L., Li R. (2017). The Chinese prescription lianhuaqingwen capsule exerts anti-influenza activity through the inhibition of viral propagation and impacts immune function. *BMC Complementary and Alternative Medicine*.

[37] Liu C. Y., Li X. Q., Cai Sq S. Q. (2010). Pharmacology and clinical research progress of lianhua qingwen capsules. *Pharm. Clin. Chin. Materia. Med.*.

[38] Lupfer C. R., Stokes K. L., Kuriakose T., Kanneganti T.-D. (2017). Deficiency of the NOD-Like receptor NLRC5 results in decreased CD8(+) T cell function and impaired viral clearance. *Journal of Virology*.

[39] Sheldrick E. L. R., Derecka K., Marshall E. (2007). Peroxisome-proliferator-activated receptors and the control of levels of prostaglandin-endoperoxide synthase 2 by arachidonic acid in the bovine uterus. *Biochemical Journal*.

[40] Song Y. L., Yao C., Yao Y. M. (2009). XueBiJing injection versus placebo for critically ill patients with severe community-acquired pneumonia: a randomized controlled trial. *Critical Care Medicine*.

[41] Liu M.-w., Su M.-x., Zhang W. (2014). Protective effect of Xuebijing injection on paraquat-induced pulmonary injury via down-regulating the expression of p38 MAPK in rats. *BMC Complementary and Alternative Medicine*.

[42] Si T.-L., Liu Q., Ren Y.-F. (2016). Enhanced anti-inflammatory effects of DHA and quercetin in lipopolysaccharide-induced RAW264.7 macrophages by inhibiting NF-*κ*B and MAPK activation. *Molecular Medicine Reports*.

[43] Zhang L.-L., Zhang H.-T., Cai Y.-Q. (2016). Anti-inflammatory effect of mesenchymal stromal cell transplantation and quercetin treatment in a rat model of experimental cerebral ischemia. *Cellular and Molecular Neurobiology*.

[44] Wang H., Song H. X., Wang D. F. (2020). Potential mechanism of Xuanfei Baidu formula in treating new coronavirus pneumonia based on network pharmacology and molecular docking. *Journal of Hainan Medical University*.

[45] Yi L., Li Z., Yuan K. (2004). Small molecules blocking the entry of severe acute respiratory syndrome coronavirus into host cells. *Journal of Virology*.

[46] Liu H., Ye F., Sun Q. (2021). Scutellaria baicalensis extract and baicalein inhibit replication of SARS-CoV-2 and its 3C-like protease in vitro. *Journal of Enzyme Inhibition and Medicinal Chemistry*.

