# Supplementary material for: Efficacy and Safety of “Three Chinese Patent Medicines and Three TCM Prescriptions” for COVID-19: A Systematic Review and Network Meta-Analysis
Source: Evid Based Complement Alternat Med. 2022 Jan 12;2022:4654793. doi: 10.1155/2022/4654793 (PMC8753255; doi:10.1155/2022/4654793)
Supplement: Supplementary Materials — provide the methodology quality of included trials and competitive drug sequencing in the study. Forest plots for nucleic acid negative rate and the disappearance rate of respiratory, gastrointestinal, and other symptoms as well as inflammatory biomarkers are also shown. They include pooled hazard ratios for competing drugs in each outcome (Table A1), specific percentage ranking in terms of competing drugs in each outcome (Table A2), methodology quality of the 10 included randomized controlled trials according to the Cochrane handbook (Table A3), rank probability of competing drugs in each outcome (Figure A1), forest plots for nucleic acid negative rate by Bayesian network meta-analysis and traditional meta-analysis (Figure A2), forest plots for disappearance rate of respiratory symptoms by Bayesian network meta-analysis and traditional meta-analysis (Figure A3), forest plots for disappearance rate of gastrointestinal symptoms by Bayesian network meta-analysis and traditional meta-analysis (Figure A4), forest plots for disappearance rate of other symptoms by Bayesian network meta-analysis and traditional meta-analysis (Figure A5), and forest plots for disappearance rate of inflammatory biomarkers by Bayesian network meta-analysis and traditional meta-analysis (Figure A6). [file 4654793.f1.docx]

**Supplemental Tables**

**Table A1.** Pooled hazard ratios for competing drugs in each outcome.

(**a**)

| **CD** | 3.67 (0.58, 23.06) | 3.90 (1.54, 10.74) | 29334775612119664.00 (362.80, 32829768451418240000000000000000000000000000.00) | 6.65 (0.68, 101.58) |
| --- | --- | --- | --- | --- |
| 0.27 (0.04, 1.72) | **JHQG** | 1.06 (0.14, 8.81) | 7960477441488945.00 (87.72, 9453887947969216000000000000000000000000000.00) | 1.82 (0.10, 50.56) |
| 0.26 (0.09, 0.65) | 0.94 (0.11, 7.36) | **LHQW** | 7448006887568072.00 (92.16, 7704129158708139000000000000000000000000000.00) | 1.71 (0.14, 30.54) |
| 0.00 (0.00, 0.00) | 0.00 (0.00, 0.01) | 0.00 (0.00, 0.01) | **XBJ** | 0.00 (0.00, 0.02) |
| 0.15 (0.01, 1.46) | 0.55 (0.02, 10.23) | 0.58 (0.03, 7.21) | 4231978237442393.00 (45.42, 2737368483106108000000000000000000000000000.00) | **XFBD** |

(**b**)

| **CD** | 2.47 (0.38, 17.87) | 3.80 (1.55, 11.26) | 0.66 (0.06, 7.88) | 5.73 (0.62, 52.15) |
| --- | --- | --- | --- | --- |
| 0.41 (0.06, 2.65) | **JHQG** | 1.48 (0.18, 16.12) | 0.24 (0.01, 5.69) | 2.33 (0.11, 42.01) |
| 0.26 (0.09, 0.64) | 0.68 (0.06, 5.44) | **LHQW** | 0.18 (0.01, 2.26) | 1.54 (0.13, 15.27) |
| 1.52 (0.13, 17.74) | 4.09 (0.18, 88.43) | 5.63 (0.44, 89.29) | **XBJ** | 9.09 (0.30, 250.72) |
| 0.17 (0.02, 1.61) | 0.43 (0.02, 8.73) | 0.65 (0.07, 7.99) | 0.11 (0.00, 3.29) | **XFBD** |

(**c**)

| **CD** | 3.01 (0.52, 16.99) | 2.83 (0.93, 8.09) | 1.70 (0.00, 626242382416.17) | 5.30 (0.73, 48.64) |
| --- | --- | --- | --- | --- |
| 0.33 (0.06, 1.91) | **JHQG** | 0.94 (0.12, 7.17) | 0.59 (0.00, 247480856987.69) | 1.79 (0.13, 29.39) |
| 0.35 (0.12, 1.08) | 1.07 (0.14, 8.44) | **LHQW** | 0.60 (0.00, 273005353688.74) | 1.89 (0.20, 22.70) |
| 0.59 (0.00, 465931813438475.56) | 1.69 (0.00, 1441956176534666.80) | 1.67 (0.00, 1253946278646998.20) | **XBJ** | 3.57 (0.00, 2957120057556373.50) |
| 0.19 (0.02, 1.37) | 0.56 (0.03, 7.85) | 0.53 (0.04, 4.93) | 0.28 (0.00, 141304349222.16) | **XFBD** |

(**d**)

| **CD** | 7.49 (0.10, 557.10) | 9.82 (1.17, 110.25) | 1.51 (0.01, 140.08) |
| --- | --- | --- | --- |
| 0.13 (0.00, 9.92) | **JHQG** | 1.30 (0.01, 190.00) | 0.20 (0.00, 115.14) |
| 0.10 (0.01, 0.85) | 0.77 (0.01, 85.94) | **LHQW** | 0.16 (0.00, 20.71) |
| 0.66 (0.01, 67.50) | 4.96 (0.01, 2717.46) | 6.42 (0.05, 1202.75) | **XBJ** |

(**e**)

| **CD** | 26.54 (4.00, 795.15) | 4.09 (0.06, 487.96) |
| --- | --- | --- |
| 0.04 (0.00, 0.25) | **LHQW** | 0.15 (0.00, 20.14) |
| 0.24 (0.00, 17.80) | 6.55 (0.05, 1892.03) | **XFBD** |

(**f**)

| **CD** | 5.24 (1.47, 24.65) | 7.17 (0.53, 143.80) |
| --- | --- | --- |
| 0.19 (0.04, 0.68) | **LHQW** | 1.37 (0.07, 31.16) |
| 0.14 (0.01, 1.89) | 0.73 (0.03, 15.33) | **XFBD** |

(**g**)

| **CD** | 4.39 (0.06, 361.73) | 2.84 (0.09, 166.40) |
| --- | --- | --- |
| 0.23 (0.00, 15.50) | **JHQG** | 0.60 (0.00, 198.66) |
| 0.35 (0.01, 10.77) | 1.66 (0.01, 401.74) | **LHQW** |

(**h**)

| **CD** | 4.32 (0.06, 282.98) | 1.18 (0.02, 70.45) |
| --- | --- | --- |
| 0.23 (0.00, 16.04) | **JHQG** | 0.28 (0.00, 108.49) |
| 0.85 (0.01, 45.34) | 3.53 (0.01, 1394.64) | **LHQW** |

(**i**)

| **CD** | 2.52 (0.13, 43.32) | 2.34 (0.16, 50.41) | 0.92 (0.05, 20.01) |
| --- | --- | --- | --- |
| 0.40 (0.02, 7.57) | **JHQG** | 1.00 (0.02, 72.61) | 0.36 (0.01, 22.36) |
| 0.43 (0.02, 6.36) | 1.00 (0.01, 62.22) | **LHQW** | 0.38 (0.01, 25.90) |
| 1.08 (0.05, 20.67) | 2.78 (0.04, 155.16) | 2.65 (0.04, 147.11) | **XFBD** |

(**j**)

| **CD** | 2.00 (0.07, 61.87) | 2.99 (0.36, 22.67) | 1.31 (0.05, 51.54) |
| --- | --- | --- | --- |
| 0.50 (0.02, 15.30) | **JHQG** | 1.40 (0.03, 66.30) | 0.74 (0.00, 95.29) |
| 0.33 (0.04, 2.75) | 0.71 (0.02, 38.41) | **LHQW** | 0.45 (0.01, 33.14) |
| 0.77 (0.02, 21.91) | 1.36 (0.01, 335.50) | 2.24 (0.03, 109.72) | **XFBD** |

(**k**)

| **CD** | 0.00 (0.00, 0.00) | 1.30 (0.03, 55.63) | 29686967808587473000000000.00 (20555.07, 27618095292662434000000000000000000000000000000000000000000.00) | 1.00 (0.01, 141.38) |
| --- | --- | --- | --- | --- |
| 1789856574186776580.00 (251.41, 2784504934693196800000000000000000000000000000000000000000.00) | **JHQG** | 2429090137669885400.00 (243.59, 4018508294677974000000000000000000000000000000000000000000.00) | 21553617645761403000000000000000000000000000000.00 (281265474672668.34, 884256367147658600000000000000000000000000000000000000000000000000000000000000000000000000000000.00) | 1812639233632638980.00 (145.17, 3734309567773049000000000000000000000000000000000000000000.00) |
| 0.77 (0.02, 32.08) | 0.00 (0.00, 0.00) | **LHQW** | 22308057588539460000000000.00 (13178.38, 25166974010588374000000000000000000000000000000000000000000.00) | 0.77 (0.00, 372.56) |
| 0.00 (0.00, 0.00) | 0.00 (0.00, 0.00) | 0.00 (0.00, 0.00) | **XBJ** | 0.00 (0.00, 0.00) |
| 1.00 (0.01, 140.99) | 0.00 (0.00, 0.01) | 1.30 (0.00, 649.31) | 30586853390320635000000000.00 (18343.47, 26934158209964124000000000000000000000000000000000000000000.00) | **XFBD** |

(**l**)

| **CD** | 1.96 (0.11, 32.59) | 11.25 (0.31, 1693.45) | 1.17 (0.01, 92.64) |
| --- | --- | --- | --- |
| 0.51 (0.03, 9.46) | **JHQG** | 5.73 (0.06, 1705.42) | 0.62 (0.00, 87.63) |
| 0.09 (0.00, 3.20) | 0.17 (0.00, 15.69) | **LHQW** | 0.09 (0.00, 32.29) |
| 0.85 (0.01, 66.94) | 1.61 (0.01, 291.32) | 10.66 (0.03, 5698.55) | **XFBD** |

(**m**)

| **CD** | 17.02 (0.24, 2581.27) | 4.95 (0.00, 11734.05) |
| --- | --- | --- |
| 0.06 (0.00, 4.13) | **LHQW** | 0.31 (0.00, 1812.11) |
| 0.20 (0.00, 489.35) | 3.24 (0.00, 42802.74) | **XFBD** |

(**n**)

| **CD** | 1.72 (0.14, 23.13) | 5.43 (1.14, 35.86) |
| --- | --- | --- |
| 0.58 (0.04, 7.38) | **JHQG** | 3.85 (0.17, 78.88) |
| 0.18 (0.03, 0.88) | 0.26 (0.01, 5.94) | **LHQW** |

(**o**)

| **CD** | 4.74 (0.37, 201.87) | 1.71 (0.07, 45.78) |
| --- | --- | --- |
| 0.21 (0.00, 2.69) | **LHQW** | 0.32 (0.00, 24.83) |
| 0.58 (0.02, 13.74) | 3.16 (0.04, 307.38) | **XFBD** |

(**p**)

| **CD** | -1.72 (-45.74, 39.60) | -12.25 (-36.46, 13.93) |
| --- | --- | --- |
| 1.72 (-39.60, 45.74) | **QFPD** | -10.31 (-57.57, 40.86) |
| 12.25 (-13.93, 36.46) | 10.31 (-40.86, 57.57) | **XBJ** |

(**q**)

| **CD** | -0.29 (-3.24, 2.43) | 0.26 (-0.74, 1.55) |
| --- | --- | --- |
| 0.29 (-2.43, 3.24) | **QFPD** | 0.58 (-2.35, 3.74) |
| -0.26 (-1.55, 0.74) | -0.58 (-3.74, 2.35) | **XBJ** |

(**r**)

| **CD** | 0.28 (0.05, 1.18) | 0.37 (0.08, 1.75) | 0.44 (0.19, 0.91) | 0.37 (0.07, 1.77) | 0.78 (0.14, 4.26) | 0.18 (0.02, 1.07) |
| --- | --- | --- | --- | --- | --- | --- |
| 3.53 (0.85, 19.19) | **HSBD** | 1.30 (0.17, 12.77) | 1.56 (0.30, 9.60) | 1.31 (0.16, 12.98) | 2.80 (0.30, 30.85) | 0.64 (0.05, 7.69) |
| 2.72 (0.57, 12.73) | 0.77 (0.08, 5.93) | **JHQG** | 1.18 (0.19, 6.36) | 1.02 (0.10, 8.90) | 2.15 (0.21, 21.32) | 0.48 (0.04, 5.29) |
| 2.27 (1.10, 5.17) | 0.64 (0.10, 3.34) | 0.85 (0.16, 5.15) | **LHQW** | 0.86 (0.14, 4.72) | 1.75 (0.28, 12.10) | 0.41 (0.05, 2.88) |
| 2.67 (0.56, 13.63) | 0.76 (0.08, 6.12) | 0.98 (0.11, 9.57) | 1.16 (0.21, 7.34) | **QFPD** | 2.14 (0.20, 22.19) | 0.47 (0.04, 5.29) |
| 1.29 (0.23, 7.12) | 0.36 (0.03, 3.39) | 0.47 (0.05, 4.72) | 0.57 (0.08, 3.58) | 0.47 (0.05, 4.89) | **XBJ** | 0.23 (0.02, 2.79) |
| 5.68 (0.94, 44.81) | 1.55 (0.13, 18.86) | 2.08 (0.19, 28.45) | 2.45 (0.35, 21.52) | 2.12 (0.19, 24.94) | 4.39 (0.36, 65.69) | **XFBD** |

(**s**)

| **CD** | 10180783059.54 (57.36, 84035589539613030000000000000000.00) | 0.48 (0.06, 2.33) | 0.50 (0.11, 4.33) | 1.69 (0.32, 11.46) |
| --- | --- | --- | --- | --- |
| 0.00 (0.00, 0.02) | **JHQG** | 0.00 (0.00, 0.01) | 0.00 (0.00, 0.01) | 0.00 (0.00, 0.03) |
| 2.08 (0.43, 16.89) | 23593849558.56 (116.96, 207609347435910950000000000000000.00) | **LHQW** | 1.04 (0.14, 25.51) | 3.62 (0.39, 67.30) |
| 2.00 (0.23, 9.20) | 19079174885.92 (94.66, 154083222140173640000000000000000.00) | 0.96 (0.04, 7.40) | **QFPD** | 3.32 (0.22, 36.48) |
| 0.59 (0.09, 3.09) | 5987419188.69 (29.14, 50720821057458350000000000000000.00) | 0.28 (0.01, 2.54) | 0.30 (0.03, 4.48) | **XBJ** |

The column treatment is compared with the row treatment, numbers in parentheses indicate 95% credible intervals, a-p and r-s used RR, p and q used MD. When the entire 95% confidence interval does not contain 0 or 1, the RR or MD is statistically significant. (a) fever, (b) cough, (c) fatigue, (d) expectoration, (e) shortness of breath, (f) chest distress, (g) rhinobyon, (h) rhinorrhea, (i) sore throat, (j) nausea, (k) diarrhea, (l) emesis, (m) inappetence, (n) muscle soreness, (o) headache, (p) CRP, (q) WBC, (r) exacerbation rate, (s) adverse reaction.

**Table A2.** Specific percentage ranking in terms of competing drugs in each outcome.

**(a)**

| **Drug** | **Rank 1** | **Rank 2** | **Rank 3** | **Rank 4** | **Rank 5** |
| --- | --- | --- | --- | --- | --- |
| CD | 0 | 0 | 0.01 | 0.09 | 0.9 |
| JHQG | 0 | 0.22 | 0.34 | 0.39 | 0.05 |
| LHQW | 0 | 0.19 | 0.46 | 0.34 | 0.01 |
| XBJ | 1 | 0 | 0 | 0 | 0 |
| XFBD | 0 | 0.59 | 0.19 | 0.18 | 0.04 |

From rank1 to 5, the drugs became less effective.

**(b)**

| **Drug** | **Rank 1** | **Rank 2** | **Rank 3** | **Rank 4** | **Rank 5** |
| --- | --- | --- | --- | --- | --- |
| CD | 0 | 0.01 | 0.09 | 0.62 | 0.28 |
| JHQG | 0.16 | 0.24 | 0.43 | 0.11 | 0.06 |
| LHQW | 0.23 | 0.5 | 0.24 | 0.02 | 0 |
| XBJ | 0.03 | 0.05 | 0.1 | 0.19 | 0.63 |
| XFBD | 0.58 | 0.2 | 0.15 | 0.05 | 0.02 |

From rank1 to 5, the drugs became less effective.

**(c)**

| **Drug** | **Rank 1** | **Rank 2** | **Rank 3** | **Rank 4** | **Rank 5** |
| --- | --- | --- | --- | --- | --- |
| CD | 0 | 0.01 | 0.07 | 0.49 | 0.44 |
| JHQG | 0.12 | 0.3 | 0.35 | 0.19 | 0.04 |
| LHQW | 0.08 | 0.3 | 0.4 | 0.21 | 0.01 |
| XBJ | 0.46 | 0.02 | 0.01 | 0.02 | 0.49 |
| XFBD | 0.33 | 0.38 | 0.17 | 0.1 | 0.02 |

From rank1 to 5, the drugs became less effective.

**(d)**

| **Drug** | **Rank 1** | **Rank 2** | **Rank 3** | **Rank 4** |
| --- | --- | --- | --- | --- |
| CD | 0 | 0.07 | 0.43 | 0.5 |
| JHQG | 0.4 | 0.33 | 0.16 | 0.1 |
| LHQW | 0.46 | 0.42 | 0.11 | 0.01 |
| XBJ | 0.13 | 0.18 | 0.3 | 0.39 |

From rank1 to 4, the drugs became less effective.

**(e)**

| **Drug** | **Rank 1** | **Rank 2** | **Rank 3** |
| --- | --- | --- | --- |
| CD | 0 | 0.2 | 0.8 |
| LHQW | 0.81 | 0.19 | 0 |
| XFBD | 0.19 | 0.61 | 0.2 |

From rank1 to 3, the drugs became less effective.

**(f)**

| **Drug** | **Rank 1** | **Rank 2** | **Rank 3** |
| --- | --- | --- | --- |
| CD | 0 | 0.07 | 0.93 |
| LHQW | 0.41 | 0.58 | 0.01 |
| XFBD | 0.59 | 0.35 | 0.06 |

From rank1 to 3, the drugs became less effective.

**(g)**

| **Drug** | **Rank 1** | **Rank 2** | **Rank 3** |
| --- | --- | --- | --- |
| CD | 0.06 | 0.37 | 0.57 |
| JHQG | 0.55 | 0.25 | 0.2 |
| LHQW | 0.39 | 0.38 | 0.23 |

From rank1 to 3, the drugs became less effective.

**(h)**

| **Drug** | **Rank 1** | **Rank 2** | **Rank 3** |
| --- | --- | --- | --- |
| CD | 0.09 | 0.5 | 0.41 |
| JHQG | 0.62 | 0.22 | 0.17 |
| LHQW | 0.29 | 0.29 | 0.43 |

From rank1 to 3, the drugs became less effective.

**(i)**

| **Drug** | **Rank 1** | **Rank 2** | **Rank 3** | **Rank 4** |
| --- | --- | --- | --- | --- |
| CD | 0.03 | 0.23 | 0.48 | 0.25 |
| JHQG | 0.4 | 0.29 | 0.16 | 0.15 |
| LHQW | 0.4 | 0.27 | 0.16 | 0.17 |
| XFBD | 0.17 | 0.2 | 0.2 | 0.43 |

From rank1 to 4, the drugs became less effective.

**(j)**

| **Drug** | **Rank 1** | **Rank 2** | **Rank 3** | **Rank 4** |
| --- | --- | --- | --- | --- |
| CD | 0.02 | 0.21 | 0.45 | 0.32 |
| JHQG | 0.33 | 0.24 | 0.18 | 0.25 |
| LHQW | 0.39 | 0.35 | 0.17 | 0.08 |
| XFBD | 0.26 | 0.19 | 0.2 | 0.35 |

From rank1 to 4, the drugs became less effective.

**(k)**

| **Drug** | **Rank 1** | **Rank 2** | **Rank 3** | **Rank 4** | **Rank 5** |
| --- | --- | --- | --- | --- | --- |
| CD | 0 | 0.22 | 0.5 | 0.28 | 0 |
| JHQG | 0 | 0 | 0 | 0 | 1 |
| LHQW | 0 | 0.41 | 0.28 | 0.3 | 0 |
| XBJ | 1 | 0 | 0 | 0 | 0 |
| XFBD | 0 | 0.37 | 0.21 | 0.41 | 0 |

From rank1 to 5, the drugs became less effective.

**(l)**

| **Drug** | **Rank 1** | **Rank 2** | **Rank 3** | **Rank 4** |
| --- | --- | --- | --- | --- |
| CD | 0.02 | 0.18 | 0.46 | 0.34 |
| JHQG | 0.17 | 0.36 | 0.26 | 0.21 |
| LHQW | 0.65 | 0.21 | 0.08 | 0.05 |
| XFBD | 0.17 | 0.24 | 0.19 | 0.4 |

From rank1 to 4, the drugs became less effective.

**(m)**

| **Drug** | **Rank 1** | **Rank 2** | **Rank 3** |
| --- | --- | --- | --- |
| CD | 0.03 | 0.34 | 0.63 |
| LHQW | 0.6 | 0.34 | 0.06 |
| XFBD | 0.37 | 0.32 | 0.31 |

From rank1 to 3, the drugs became less effective.

**(n)**

| **Drug** | **Rank 1** | **Rank 2** | **Rank 3** |
| --- | --- | --- | --- |
| CD | 0.01 | 0.36 | 0.63 |
| JHQG | 0.18 | 0.46 | 0.35 |
| LHQW | 0.81 | 0.18 | 0.01 |

From rank1 to 3, the drugs became less effective.

**(o)**

| **Drug** | **Rank 1** | **Rank 2** | **Rank 3** |
| --- | --- | --- | --- |
| CD | 0.04 | 0.41 | 0.55 |
| LHQW | 0.66 | 0.24 | 0.1 |
| XFBD | 0.29 | 0.35 | 0.36 |

From rank1 to 3, the drugs became less effective.

**(p)**

| **Drug** | **Rank 1** | **Rank 2** | **Rank 3** |
| --- | --- | --- | --- |
| CD | 0.46 | 0.47 | 0.07 |
| QFPD | 0.44 | 0.26 | 0.3 |
| XBJ | 0.1 | 0.27 | 0.63 |

From rank1 to 3, the drugs became less effective.

**(q)**

| **Drug** | **Rank 1** | **Rank 2** | **Rank 3** |
| --- | --- | --- | --- |
| CD | 0.15 | 0.55 | 0.31 |
| QFPD | 0.32 | 0.12 | 0.56 |
| XBJ | 0.54 | 0.33 | 0.13 |

From rank1 to 3, the drugs became less effective.

**(r)**

| **Drug** | **Rank 1** | **Rank 2** | **Rank 3** | **Rank 4** | **Rank 5** | **Rank 6** | **Rank 7** |
| --- | --- | --- | --- | --- | --- | --- | --- |
| CD | 0.49 | 0.41 | 0.08 | 0.01 | 0.00 | 0.00 | 0.00 |
| HSBD | 0.02 | 0.06 | 0.11 | 0.14 | 0.19 | 0.27 | 0.21 |
| JHQG | 0.06 | 0.08 | 0.17 | 0.17 | 0.22 | 0.18 | 0.12 |
| LHQW | 0.01 | 0.08 | 0.27 | 0.32 | 0.20 | 0.10 | 0.02 |
| QFPD | 0.06 | 0.10 | 0.16 | 0.17 | 0.19 | 0.19 | 0.12 |
| XBJ | 0.34 | 0.24 | 0.14 | 0.10 | 0.08 | 0.07 | 0.02 |
| XFBD | 0.02 | 0.03 | 0.06 | 0.09 | 0.11 | 0.19 | 0.50 |

From rank 1 to 7, the drugs became more effective.

**(s)**

| **Drug** | **Rank 1** | **Rank 2** | **Rank 3** | **Rank 4** | **Rank 5** |
| --- | --- | --- | --- | --- | --- |
| CD | 0 | 0.17 | 0.64 | 0.17 | 0.02 |
| JHQG | 1 | 0 | 0 | 0 | 0 |
| LHQW | 0 | 0.04 | 0.08 | 0.39 | 0.49 |
| QFPD | 0 | 0.08 | 0.12 | 0.35 | 0.45 |
| XBJ | 0 | 0.71 | 0.16 | 0.09 | 0.04 |

From rank1 to 5, the drugs became less effective.

1. fever; (b) cough; (c) fatigue; (d) expectoration; (e) shortness of breath; (f) chest distress; (g) rhinobyon; (h) rhinorrhea; (i) sore throat; (j) nausea; (k) diarrhea; (l) emesis; (m) inappetence; (n) muscle soreness; (o) headache; (p) CRP; (q) WBC; (r) exacerbation rate; (s) adverse reaction.

**Table A3.** Methodology quality of the 10 included randomized controlled trials according to the Cochrane handbook.

| References | A | B | C | D | E | F |
| --- | --- | --- | --- | --- | --- | --- |
| Chen et al., 2020 | ? | ? | ? | - | - | - |
| Duan et al., 2020 | - | ? | ? | - | - | - |
| Hu et al., 2020 | - | ? | ? | - | - | - |
| Li and Zhang et al., 2020 | ? | ? | ? | - | - | - |
| Luo et al., 2021 | - | - | ? | - | - | - |
| Wen et al, 2020 | - | ? | ? | - | - | - |
| Xiong et al., 2020 | - | ? | ? | - | - | - |
| Yu et al., 2020 | - | ? | ? | - | - | - |
| Zhang et al., 2020 | ? | ? | ? | - | - | - |
| Zhao et al., 2021 | - | ? | ? | - | - | - |

A, adequate sequence generation; B, allocation concealment; C, blinding; D, incomplete outcome data; E, selective reporting; F, other bias; +, high risk; -, low risk; ?, unclear.

**Table A4.** Results of quality assessment of the 8 included retrospective studies by using NOS scale.

| Items | References (scores) | | | | | | | |
| --- | --- | --- | --- | --- | --- | --- | --- | --- |
|  | Cheng et al., 2020 | Fang et al., 2020 | Guo et al., 2020 | Li  et al., 2020 | Lv  et al., 2020 | Xu  et al., 2020 | Yao  et al., 2020 | Zeng  et al., 2020 |
| **The selection of participants (4 scores)** |  |  |  |  |  |  |  |  |
| Representativeness of the exposure group | 1 | 1 | 1 | 1 | 1 | 1 | 1 | 1 |
| Selection of non-exposed groups | 1 | 1 | 1 | 1 | 1 | 1 | 1 | 1 |
| Determination of exposure factors | 1 | 1 | 1 | 1 | 1 | 1 | 1 | 1 |
| There were no outcome indicators to look for at the start of the study | 0 | 0 | 0 | 0 | 0 | 0 | 0 | 0 |
| **Intergroup comparability (2 scores)** |  |  |  |  |  |  |  |  |
| Comparability between exposed and non-exposed groups was considered in the design and statistical analysis | 2 | 2 | 2 | 2 | 2 | 2 | 2 | 2 |
| **Outcome measurement (3 scores)** |  |  |  |  |  |  |  |  |
| Evaluation of outcomes | 1 | 1 | 1 | 1 | 1 | 1 | 1 | 1 |
| Follow-up was long enough | 0 | 0 | 0 | 0 | 0 | 0 | 0 | 0 |
| Complete follow-up of the exposure group and the exposure group | 0 | 0 | 0 | 0 | 0 | 0 | 0 | 0 |
| **Total** | 6 | 6 | 6 | 6 | 6 | 6 | 6 | 6 |

**Supplemental Figures**

(**a**)


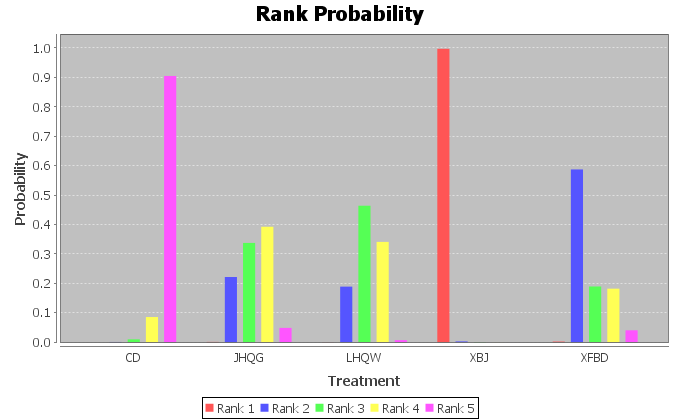


(**b**)


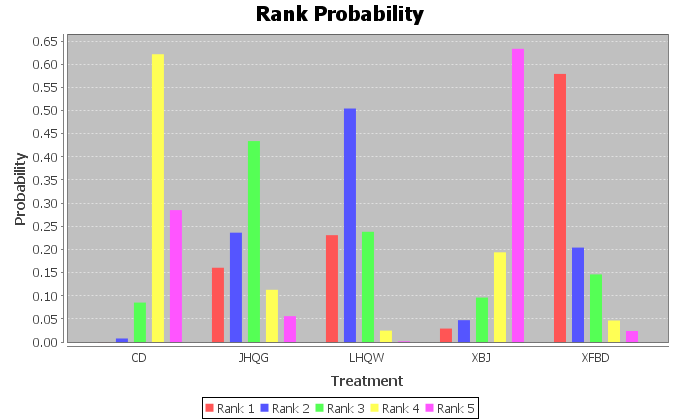


(**c**)


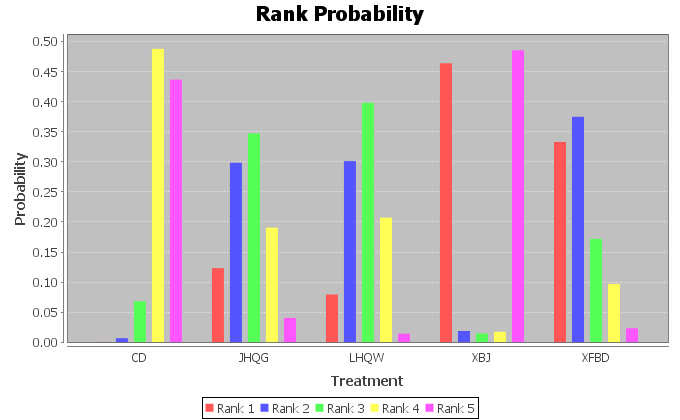


(**d**)


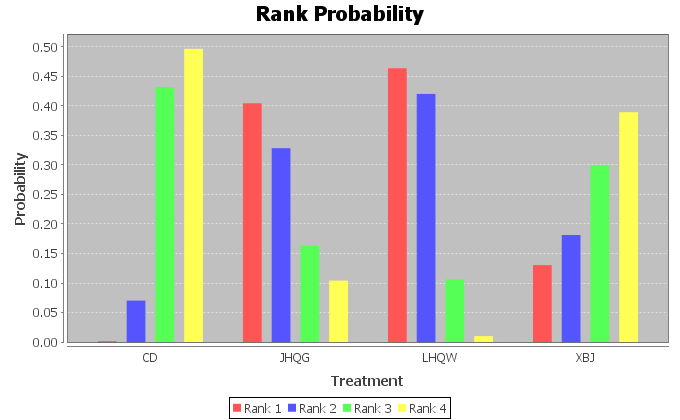


(**e**)


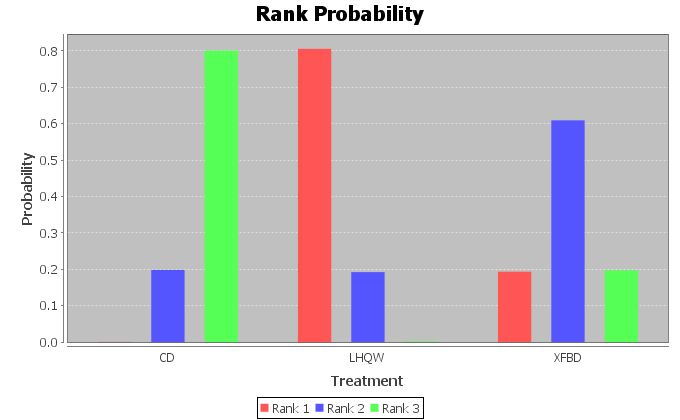


(**f**)


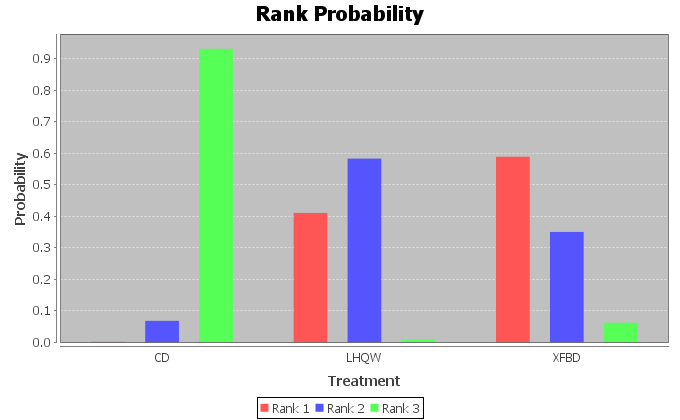


(**g**)


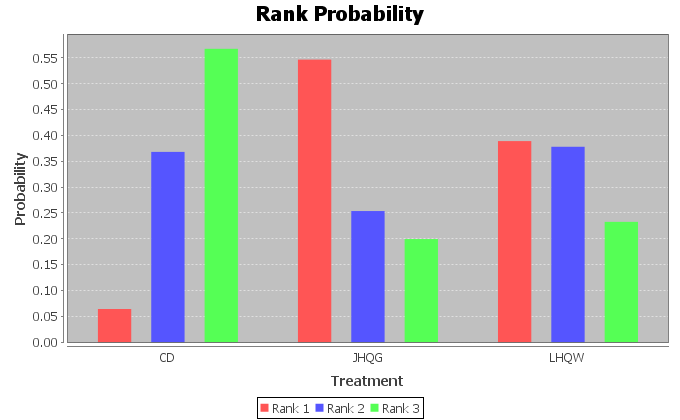


(**h**)


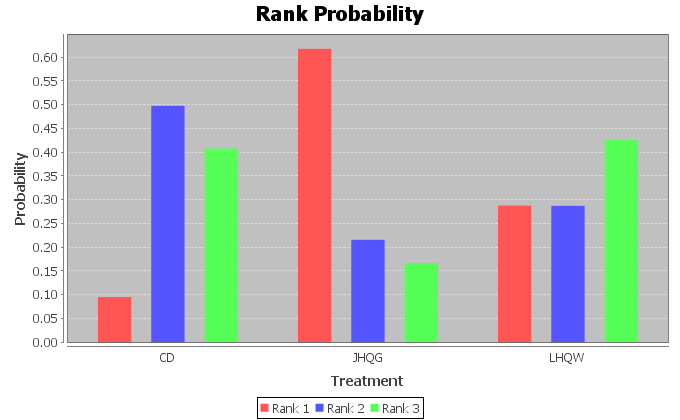


(**i**)


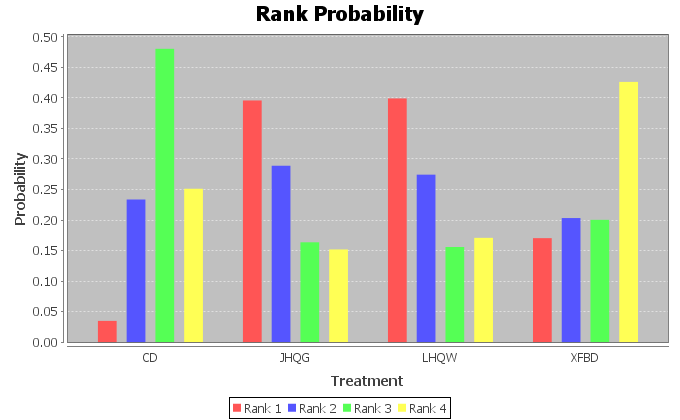


(**j**)


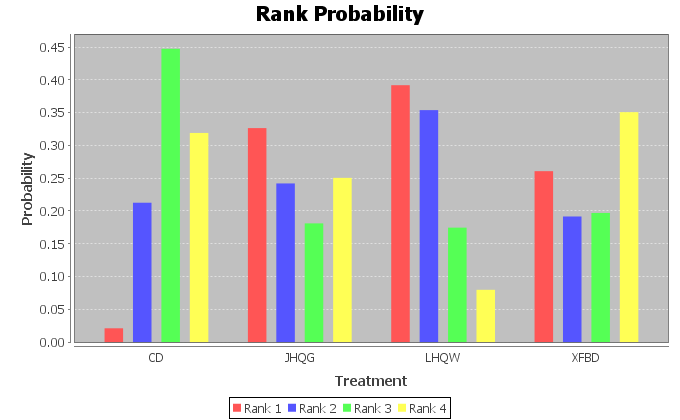


(**k**)


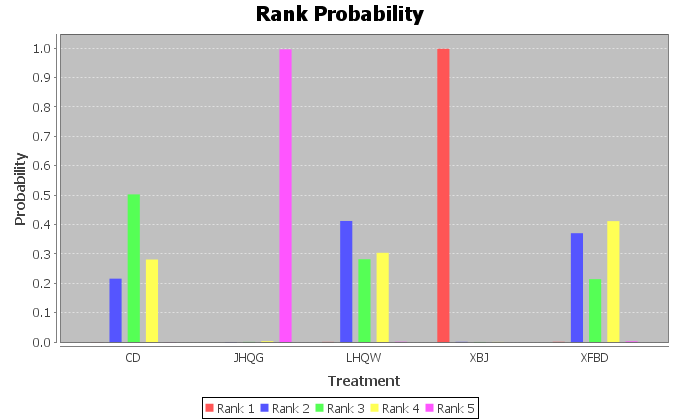


(**l**)


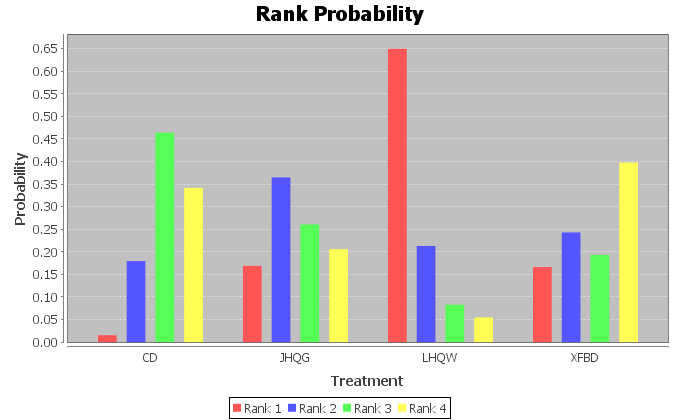


(**m**)


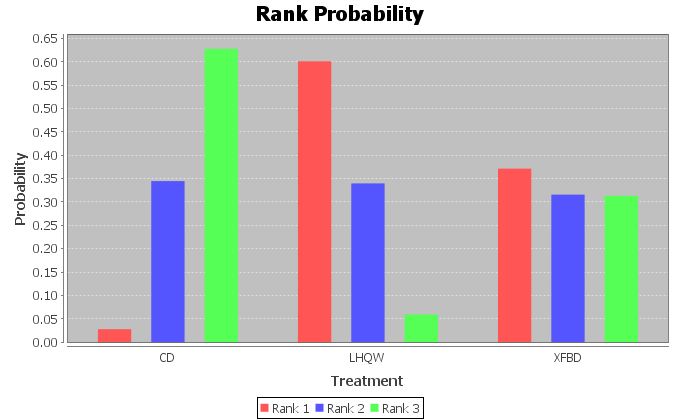


(**n**)


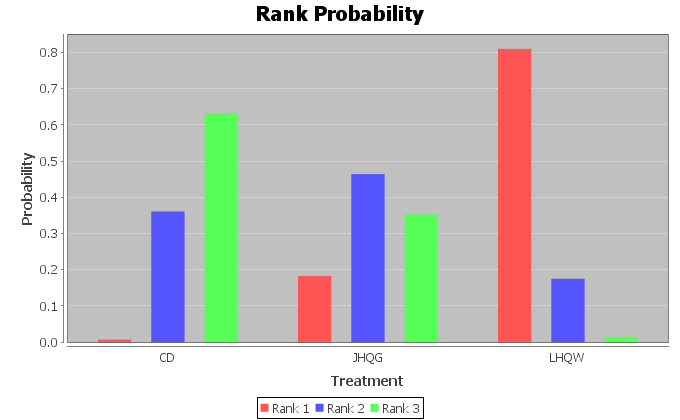


(**o**)


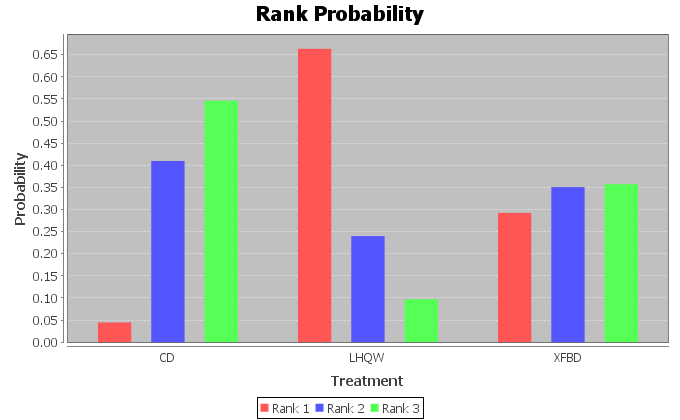


(**p**)


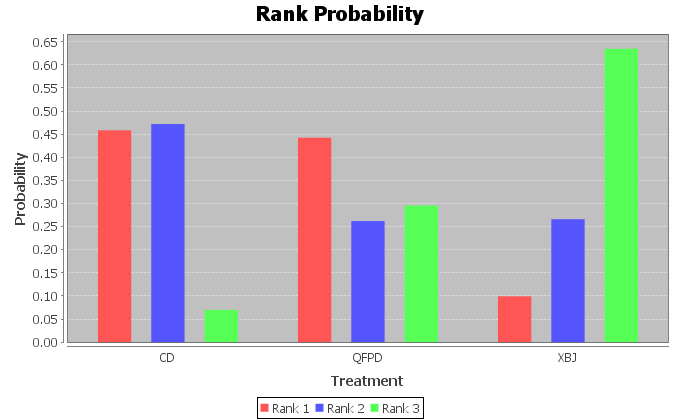


(**q**)


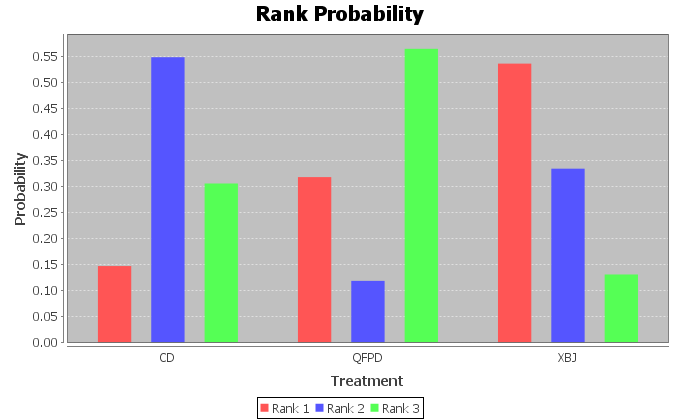


(**r**)


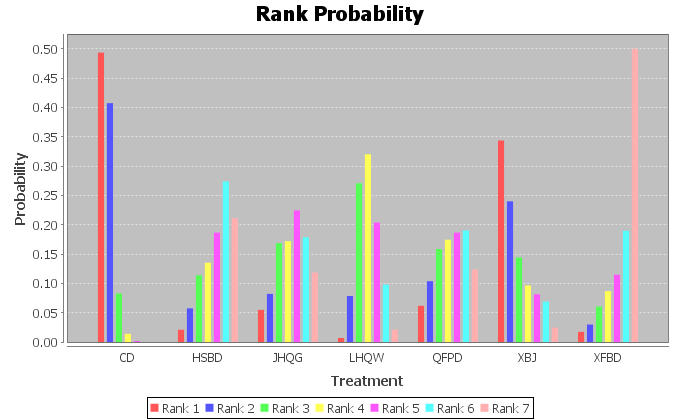


(**s**)


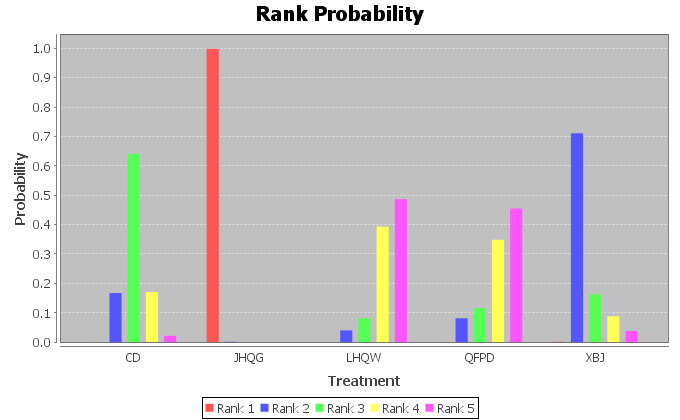


**Figure A1. Rank probability of competing drugs in each outcome.** (a) fever, (b) cough (c) fatigue, (d) expectoration; (e) shortness of breath; (f) chest distress; (g) rhinobyon; (h) rhinorrhea; (i) sore throat; (j) nausea; (k) diarrhea; (l) emesis; (m) inappetence; (n) muscle soreness; (o) headache; (p) CRP; (q) WBC; (r) exacerbation rate; (s) adverse reaction.

**Figure A2.** Forest plots for nucleic acid negative rate by Bayesian network meta-analysis and traditional meta-analysis.

(**a**)

(**b**)

(**c**)

(**d**)

(**e**)

(**f**)

**Figure A3.** Forest plots for disappearance rate of respiratory symptoms by Bayesian network meta-analysis and traditional meta-analysis. (**a**) expectoration, (**b**) shortness of breath, (**c**) chest distress, (**d**) rhinobyon, (**e**) rhinorrhea, (**f**) sore throat.

(**a**)

(**b**)

(**c**)

(**d**)

**Figure A4.** Forest plots for disappearance rate of gastrointestinal symptoms by Bayesian network meta-analysis and traditional meta-analysis. (**a**) nausea, (**b**) diarrhea, (**c**) emesis, (**d**) inappetence.

(**a**)

(**b**)

**Figure A5.** Forest plots for disappearance rate of other symptoms by Bayesian network meta-analysis and traditional meta-analysis. (**a**) muscle soreness, (**b**) headache.

(**a**)

(**b**)

**Figure A6.** Forest plots for disappearance rate of inflammatory biomarkers by Bayesian network meta-analysis and traditional meta-analysis. (**a**) CRP, (**b**) WBC.
